# Replacing anaerobic fermentation with reverse β-oxidation enables growth-coupled evolution for enhanced fatty-alcohol production

**DOI:** 10.1038/s41467-026-78481-1

**Published:** 2026-10-14

**Authors:** Bridgie Cawthon, Aidan E. Cowan, Zen Yoshikawa, Kenneth Low, Justin Baerwald, Nawa Raj Baral, Matthew Incha, Jennifer Gin, Yan Chen, Christopher J. Petzold, Edward E. K. Baidoo, Emine A. Turumtay, Corinne D. Scown, Adam M. Feist, Jay D. Keasling

**Affiliations:** 1 Joint BioEnergy Institute, Lawrence Berkeley National Laboratory, Emeryville, CA USA; 2 Department of Chemical and Biomolecular Engineering, University of California, Berkeley, Berkeley, CA USA; 3 Department of Bioresource and Environmental Security, Sandia National Laboratories, Livermore, CA USA; 4 Energy & Biosciences Institute, University of California, Berkeley, Berkeley, CA USA; 5 Department of Bioengineering, University of California, San Diego, La Jolla, CA USA; 6 The Novo Nordisk Foundation Center for Biosustainability, Technical University of Denmark, Kongens Lyngby, Denmark; 7 QB3 Institute, University of California, Berkeley, Berkeley, CA USA; 8 Department of Bioengineering, University of California, Berkeley, Berkeley, CA USA

**Keywords:** Metabolic engineering, Industrial microbiology, Environmental biotechnology

## Abstract

Engineering robust fermentation hosts remains a barrier to scalable biomanufacturing. In this work we replace canonical mixed-acid fermentation in *Escherichia coli* with a redox‑balanced reverse β‑oxidation (rBOX) pathway that produces medium‑chain fatty alcohols and is bioenergetically equivalent to ethanol fermentation. This synthetic fermentation restores anaerobic growth, tightly coupling fitness to product formation and enabling adaptive laboratory evolution that selects for mutations increasing rBOX flux. Evolved strains increase maximum productivity fourfold (110 ± 0.15 mg/L/hr) and titer more than threefold up to 4.0 ± 0.28 g/L compared to the unevolved strain. Proteomic analyses of evolved strains indicate differential expression of enzymes at key metabolic nodes and activation of latent genes with functions overlapping those of rBOX enzymes. These results establish redox-balanced growth coupling as a powerful tool for strain improvement and demonstrate rBOX as a practical synthetic fermentation pathway.

## Abstract (web summary)

Engineering robust fermentation hosts remains a barrier to scalable biomanufacturing. Here the authors link *E. coli* growth to fatty-alcohol production via the reverse β‑oxidation pathway, enabling self-improvement of alcohol production through bacterial evolution.

## Introduction

Microbial fermentation offers alternative production routes for molecules typically derived from petroleum, including fuels^1,2^, commodity chemicals^3^, materials^4^, food additives^5^, and drugs^6,7^. In general, microbial bioproduction is more sustainable and less carbon-intensive than conventional petrochemical processes^2,8,9^. Microbial bioproduction relies on the use of organic feedstocks, generally sugars, derived from starch or plant biomass^7,10^. By utilizing plant matter or other CO_2_-derived carbon for the production of chemicals, bioproduction enables circular uses of carbon and energy. However, only a limited range of biomolecules have been produced at scales sufficient to displace established petrochemical processes^11,12^. A major barrier to wider adoption is the substantial cost and complexity associated with the development of bioproduction strains capable of high titer, rate, and yield production^13,14^. The dominant method of improving bioproduction strains is rational design through laborious and costly iterative Design-Build-Test-Learn (DBTL) cycles.

An emerging field of research into microbial growth coupling seeks to facilitate DBTL cycles by tying the rate of cell division to a desired trait^15,16^ such as enzymatic activity^17,18^ or uptake rate of an alternative carbon source^19–21^. Generally, this is accomplished by engineering a deficiency in a key metabolite or cofactor, and then complementing this deficiency by the activity of a heterologous pathway or enzymatic module capable of regenerating the missing metabolite or cofactor^22–24^. This reduces the reliance on costly, time- and resource-intensive analytical or biochemical techniques by allowing growth to serve as a proxy for fitness, enabling faster DBTL cycles^15,25^ or high-throughput selection schemes for large DNA libraries^26–30^. Furthermore, growth coupling can be implemented in production strains to ensure genetic stability and directly enhance production phenotypes^31–33^.

When growth coupling imposes stringent selection such that alternative routes to improved growth are limited, adaptive laboratory evolution (ALE) could be used to improve strain performance^34,35^. ALE relies on iterative passaging under a defined selection regime to enrich spontaneous mutations that improve fitness under those conditions, including mutations that mitigate product toxicity or compensate for fitness costs introduced by strain engineering, potentially increasing pathway flux. Such selection schemes can, but need not, be implemented through metabolic growth coupling. For example, ALE has been particularly useful in the context of one-carbon (C1) utilization, where strains are engineered so that growth is directly coupled to some amount of C1 assimilation, such that serial passaging selects for mutations that improve expression of introduced modules and reconfigure central metabolism to support the new carbon entry point^19–21^.

Some studies have demonstrated ALE as a useful technique to enable the redirection of flux to enhance production of a native metabolite in a growth-coupled manner^36,37^. However, few studies have been able to harness ALE to improve the flux of a growth-coupled, non-native pathway^38^. This may be due to the relative difficulty of establishing robust growth-coupled phenotypes, where productivity remains durably linked to fitness over timescales relevant to strain evolution^39^. Instability can arise from the inherent trade-off between flux to a given bioproduct and to biomass, where cells diverting a high amount of flux to a product generally grow more slowly or to a lower terminal density than unengineered cells due to conservation of mass^38,40–42^. This can result in growth-coupled strains that are metastable in their growth-coupled phenotype, with potential to evolve mechanisms around selection to reach metabolic states that restore maximum flux to biomass^43–45^. Furthermore, growth-coupled selection strains are often poor production chassis because the coupling logic frequently selects for conversion of the coupled metabolite into biomass precursors rather than its extracellular accumulation, and because the genetic modifications used to impose coupling can introduce substantial growth and production penalties under process-relevant conditions^22,23,44^.

We sought to generate a robustly growth-coupled strain that could be used for long-term ALE, with the end goal of using the resulting evolved strain as a bioproduction chassis directly. Using ALE to improve the production of the growth-coupled product, and thus growth of the strain producing it, would demonstrate a less labor-intensive method of strain improvement, where growth coupling could be used as a hands-off means of strain development, bypassing laborious DBTL cycles^34^. Furthermore, using ALE for strain improvement would allow us to identify and incorporate causal host strain mutations that benefit bioproduction rather than taking a strictly pathway-focused approach targeting only enzyme-specific mutations. This technique would generate a strain that self-improves through continued bioproduction, enabling a stable bioproduction chassis with minimal expense and further potential for improvement through continued growth.

To ground our growth-coupled ALE strategy in a proven biological principle, we looked to anaerobic fermentation pathways, systems where flux to product is intrinsically tied to the requirements of growth. Biosynthesis of anaerobic fermentation products evolved to oxidize NADH and regenerate the NAD^+^ required as a cofactor for catabolic pathways under anaerobic conditions. Many of the high-volume bioproducts that have reached industrial scale are anaerobic fermentation products, including ethanol^46^ (≈10^11 ^kg/yr), lactic acid^47^ (≈10^9 ^kg/yr), and succinic acid^48^ (≈10^7 ^kg/yr)^4,49^, demonstrating the power of the selective pressure exerted on these pathways to make them incredibly efficient over evolutionary time. Replacing native fermentation pathways with a synthetic pathway that produces a value-added product could create a stably growth-coupled strain that can undergo adaptive evolution to yield an improved bioproduction chassis.

However, the number of pathways that are natively used for anaerobic fermentation is relatively limited. These pathways must produce sufficient flux to enable balancing the substantial redox requirements of central metabolism^50,51^. Additionally, these pathways must satisfy the strict bioenergetic constraints of metabolic stoichiometry, consuming enough NADH per unit product to stoichiometrically rebalance metabolism while not relying upon cofactors that may be limited under anaerobic conditions, such as ATP^52–54^. While these requirements substantially limit the number of bioproducts that can serve as anaerobic fermentation products, they do not preclude the existence of yet-unexplored pathways of biotechnological interest that could serve as primary anaerobic fermentation pathways^55^.

We identify a reverse β-oxidation (rBOX) pathway for fatty alcohol production that is bioenergetically equivalent to ethanol fermentation as a synthetic pathway that meets the bioenergetic requirements of anaerobic fermentation. rBOX is a biosynthetic pathway involving the elongation of acyl chains by Claisen condensation with acetyl-CoA (or other acyl-CoAs), followed by sequential reduction and dehydration of the resulting β-keto group and saturation of the resulting enoyl double bond, effectively operating as the reverse of fatty acid β-oxidation^56,57^. rBOX is an advantageous strategy for producing acyl-CoA-derived biomolecules, as it bypasses the ATP-dependent activation of acetyl-CoA to malonyl-CoA required in canonical fatty acid biosynthesis and instead utilizes acetyl-CoA directly as the two-carbon extender unit, thereby enabling a more energy-efficient elongation process^58^. rBOX holds significant promise for sustainable biosynthesis of chemicals. However, its efficient implementation demands precise control over enzyme expression levels, identification and relief of pathway bottlenecks, and careful management of redox balance and precursor metabolite availability^59–61^. Despite its promise, implementing reverse β-oxidation at titers, rates, and yields required for scalability remains a significant metabolic-engineering challenge^61^. Furthermore, an outstanding challenge remains in adapting rBOX production schemes to minimal media and anaerobic conditions, both of which are required to achieve maximum cost efficiency for commodity production.

Herein, we replace endogenous fermentation pathways in *Escherichia coli* with rBOX, enabling a growth-coupled phenotype where catabolism of glucose under anaerobic conditions is entirely dependent on high flux through an rBOX pathway producing fatty alcohols. We exploit this growth-coupled strategy by employing ALE to improve the growth and production of an rBOX strain in an untargeted manner. This approach requires no engineering interventions beyond iterative culturing and yields a strain with roughly a fourfold increase in fatty-alcohol productivity and threefold increase in maximum titer in anaerobic fed-batch fermentation using minimal medium.

## Results

### Establishing growth-coupled phenotype based on complementation of fermentation deficit

**Fig. 1:**
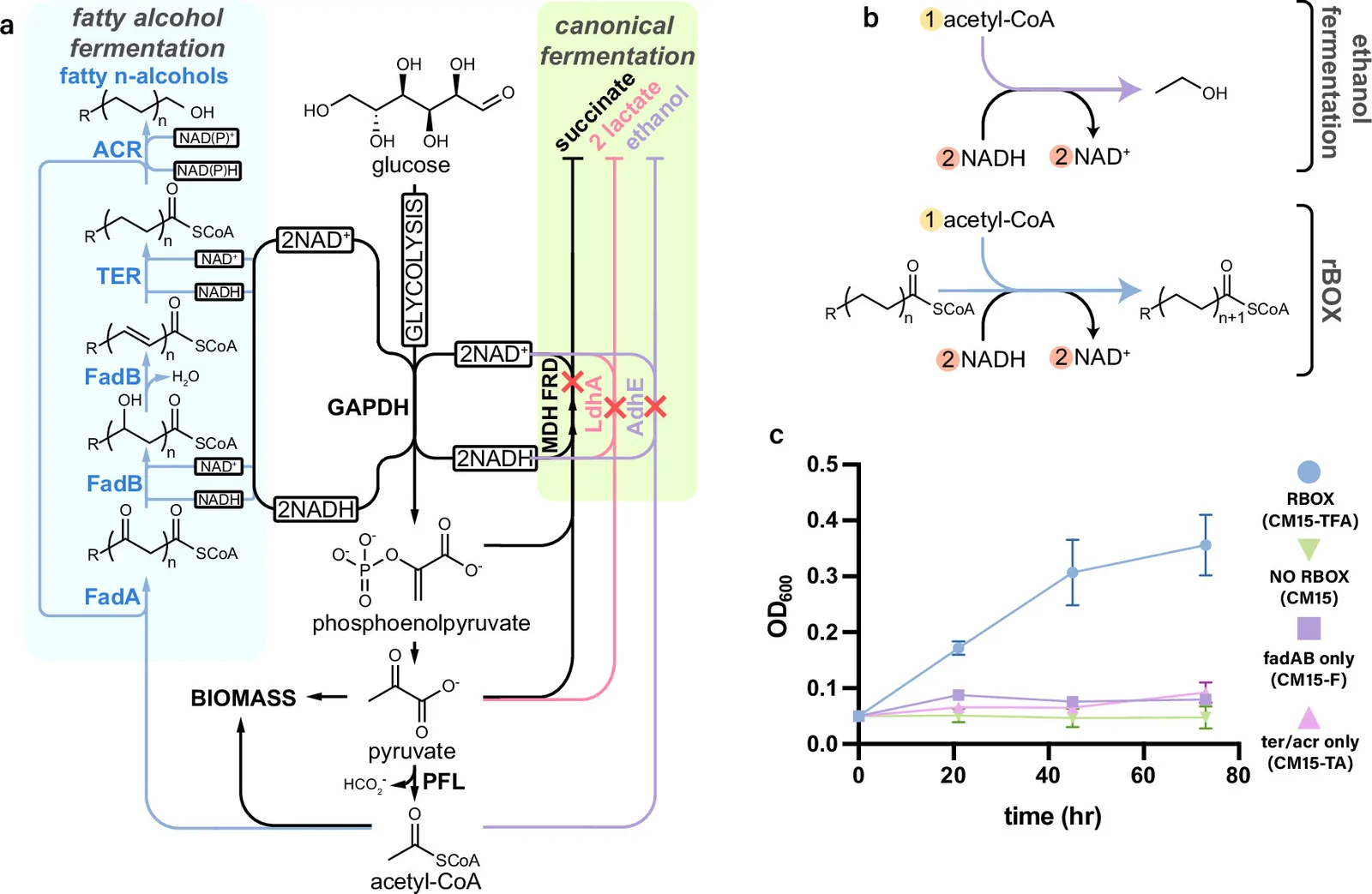
Establishing growth-coupled phenotype based on complementation of fermentation deficit. **a** Schematic diagram illustrating metabolic role of fatty-alcohol biosynthesis through rBOX in a fermentation-deficient strain of *E. coli* (CM15). Canonical fermentation pathways (green highlight) are blocked (red X) and halt the regeneration of NAD^+^ required as a cofactor of glyceraldehyde 3-phosphate dehydrogenase (GAPDH), thus preventing glycolysis from proceeding. Fatty-alcohol fermentation through rBOX (blue highlight) is depicted showing all key intermediates and genes overexpressed in *E. coli*. This rBOX pathway allows for regeneration of NAD^+^, thus complementing the fermentation deficit of CM15. **b** Comparison of the stoichiometry of rBOX and ethanol fermentation. Ethanol fermentation and rBOX are stoichiometrically equivalent in terms of acetyl-CoA (yellow highlight) and NADH (orange highlight) required for production of one ethanol or one elongation of a fatty acyl-CoA. **c** Growth of CM15 strains expressing the complete or partial rBOX pathway under anaerobic conditions in M9 minimal medium containing 2% glucose (*n* = 3). Only CM15 expressing the entire rBOX pathway was able to grow. Data represent the mean ± s.d. of three biological replicates. Source data are provided as a Source Data File.

Numerous studies have examined the effects of fermentation deficits in *E. coli*^28,56,62^. We chose to use the strain CM15^56^ as a fermentation-deficient base that we could engineer to utilize rBOX to overcome its metabolic deficit under anaerobic conditions. CM15 contains deletions in the genes *adhE* and *ldhA*, which are required for anaerobic growth on glucose (Fig. 1a)^28^. It also carries deletions in the *frdABCD* operon to block the use of fumarate as a terminal electron acceptor. Additional knockouts in *ackA*, *pta*, and *poxB* prevent acetate overflow metabolism and retain more carbon for bioproduction. CM15 also contains key deletions in *fadR* and *fadE* to block the native β-oxidation pathway.

Strains lacking *adhE* and *ldhA* cannot grow anaerobically on glucose^28,63^. However, this defect can be complemented by the expression of AdhE alone to enable ethanol fermentation^28^. Building upon this principle, we sought to complement the fermentation deficit of CM15 by introducing rBOX, which is bioenergetically equivalent to ethanol fermentation in terms of acetyl-CoA and reducing equivalent requirement per elongation (Fig. 1b). To enable the complementation of CM15’s fermentation deficit with rBOX, a two plasmid system was constructed to express the genes necessary for rBOX. The first plasmid, named pBbA6a_fadAB, overexpressed the endogenous *E. coli* genes *fadA* and *fadB*. In the context of rBOX, the enzyme FadA functions as a 3-ketoacyl-CoA thiolase, condensing extender acetyl-CoA units with the elongating acyl chain by Claisen condensation (Fig. 1a). FadB is a multifunctional enzyme capable of reversibly catalyzing the reduction of the β-keto functionality, generated by the activity of FadA, as well as subsequently dehydrating the resulting β-hydroxy functional group (Fig. 1a). A second plasmid, named pSeva_TER_ACR, encodes the genes for the subsequent reactions: trans-2-enoyl-CoA reductase from *Treponema denticola* (TER)^56^ and acyl-CoA reductase from *Marinobacter aquaeolei* VT8 (ACR)^64^. TER catalyzes the reversible reduction of the enoyl-CoA intermediate, and ACR catalyzes the chain-termination reaction to release the final fatty-alcohol product (Fig. 1a).

Plasmids pSeva_TER_ACR and pBbA6a_fadAB were transformed individually into CM15, generating strains CM15-TA (containing pSeva_TER_ACR) and CM15-F (containing pBbA6a_fadAB) and together generating strain CM15-TFA (TER, fadAB and ACR). Each strain, as well as the base CM15, was assayed for growth under anaerobic conditions. CM15-TFA was able to grow over the course of the incubation period while CM15, CM15-TA and CM15-F did not show any significant growth, demonstrating a successful complementation of fermentation deficiency (Fig. 1b). These results indicate that the overexpression of the entire rBOX pathway was able to complement the deficit to fermentative metabolism to allow for anaerobic growth of the strain, thus enabling a growth-coupled phenotype that could subsequently be exploited by ALE to improve the growth and productivity of the strain.

### Adaptive laboratory evolution of rBOX-complemented fermentation-deficient strain

**Fig. 2:**
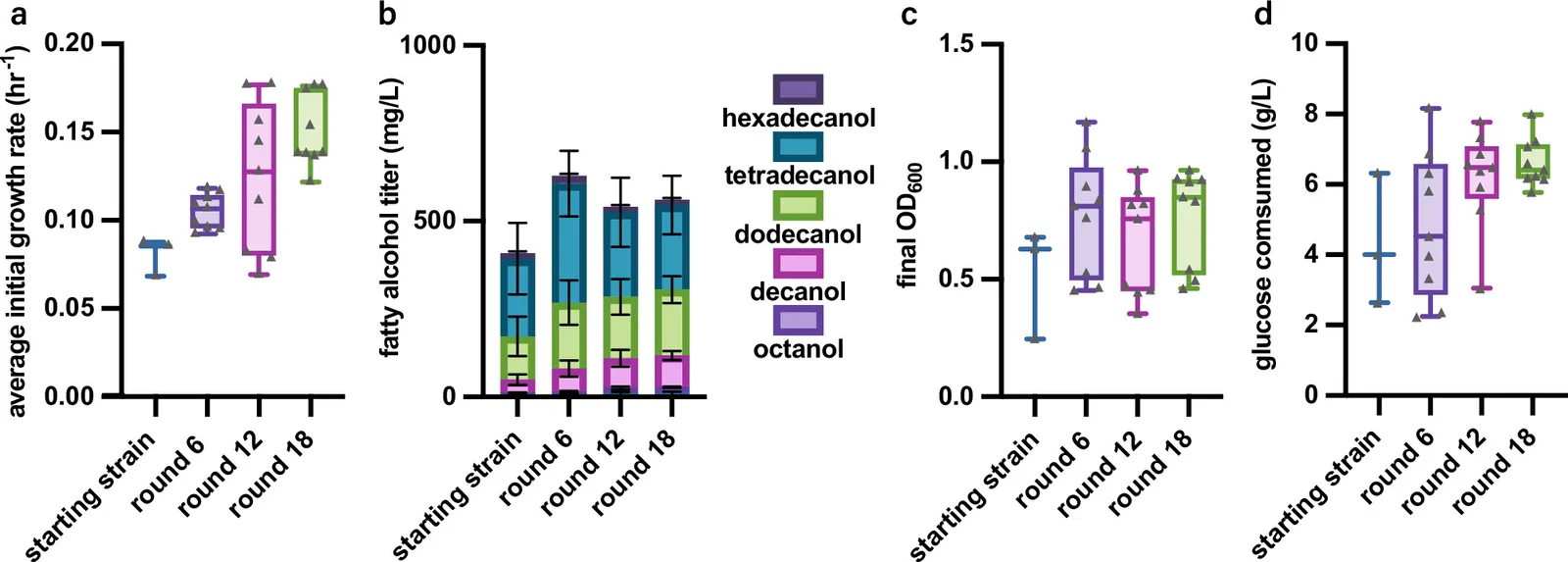
Adaptive laboratory evolution of CM15-TFA. **a** Growth rate of individual clones isolated from various rounds of evolution showing that clones isolated from later rounds of ALE had faster growth rates (starting strain *n* = 3, evolved isolates *n* = 9). **b** Fatty-alcohol titer produced by the individual clones isolated from each round of ALE after a 3-day fermentation, showing that the amount of fatty alcohols produced was approximately the same in this culture format (starting strain *n* = 3, evolved isolates *n* = 9). **c** Final optical density of isolates from round 6, 12, and 18 of ALE (*n* = 9) compared to the starting CM15-TFA strain (*n* = 3). **d** Glucose consumed in each culture of isolates from round 6, 12, and 18 of ALE (starting concentration 20 g/L) (*n* = 9) compared to the starting CM15-TFA strain (*n *= 3). Data represent the mean ± s.d. of biological replicates. In **a**, **c,** and **d**, box plots show the median (center line), 25th to 75th percentiles (box), and minimum to maximum values (whiskers); points represent biological replicates. Source data are provided as a Source Data File.

With anaerobic growth coupling established, we next sought to exploit this phenomenon to select for variants with better bioproduction metrics using ALE. Three independently growing cultures of CM15-TFA were iteratively passaged anaerobically in minimal M9 medium containing glucose for 18 rounds of evolution. After every 6 rounds of passaging, individual clones were isolated from the population and assayed for improved anaerobic growth. As the cultures adapted to the use of rBOX as a fermentation pathway through continued anaerobic growth on glucose, the growth rate of the individual clones assayed at each round increased (Fig. 2a).

To determine if the ability to produce fatty alcohols had improved over the course of the ALE, and to identify the best producing isolate, we also quantified the fatty-alcohol fermentation profile of each clone at the endpoint of a 3-day fermentation. Over the course of the adaptive evolution the strains maintained their ability to produce fatty alcohols. Accounting for variability between strains, the evolved strains produced similar overall titers of fatty alcohols compared to the starting strain (Fig. 2b). The strains also grew to similar final optical densities (Fig. 2c).

We assayed the glucose consumption in each fermentation to see if the cultures had been limited by feedstock availability and found that all the cultures had only consumed a fraction of the available 20 g/L starting concentration of glucose (Fig. 2d). This indicated that the small-scale fermentations did not provide the ideal conditions for high-density growth and optimal production, and that simply adding more glucose in this culture format was unlikely to allow for improved growth and production. Accordingly, we moved on to test the starting strain and a well-performing evolved strain in fed-batch fermentation, where additional factors limiting strain growth could be remedied to allow for optimal growth and therefore production. We chose to assay the strain from ALE passage round 18 that produced the highest titer and grew the best in small-scale fermentations. This strain was from the second ALE replicate, and we named it CM15-TFA_E2R18_ (E2 denoting evolution replicate 2 and R18 denoting passage round 18).

### Anaerobic, fed-batch fermentation of CM15-TFA and evolved CM15-TFA_E2R18_ strains

To assay the fermentation of fatty alcohols by CM15-TFA strains under controlled conditions we employed fed-batch fermentation in pH-controlled bioreactors. We used a continuous nitrogen sparge to ensure anaerobic conditions and cultured both the original CM15-TFA as well as the fastest-growing evolved strain CM15-TFA_E2R18_ in 500 mL of M9 minimal medium containing 20 g/L glucose. The pH was regulated by the addition of ammonium hydroxide, and 2.5 g/L of glucose was fed each day. Samples were taken periodically to monitor OD_600_ and fatty alcohol production.

**Fig. 3:**
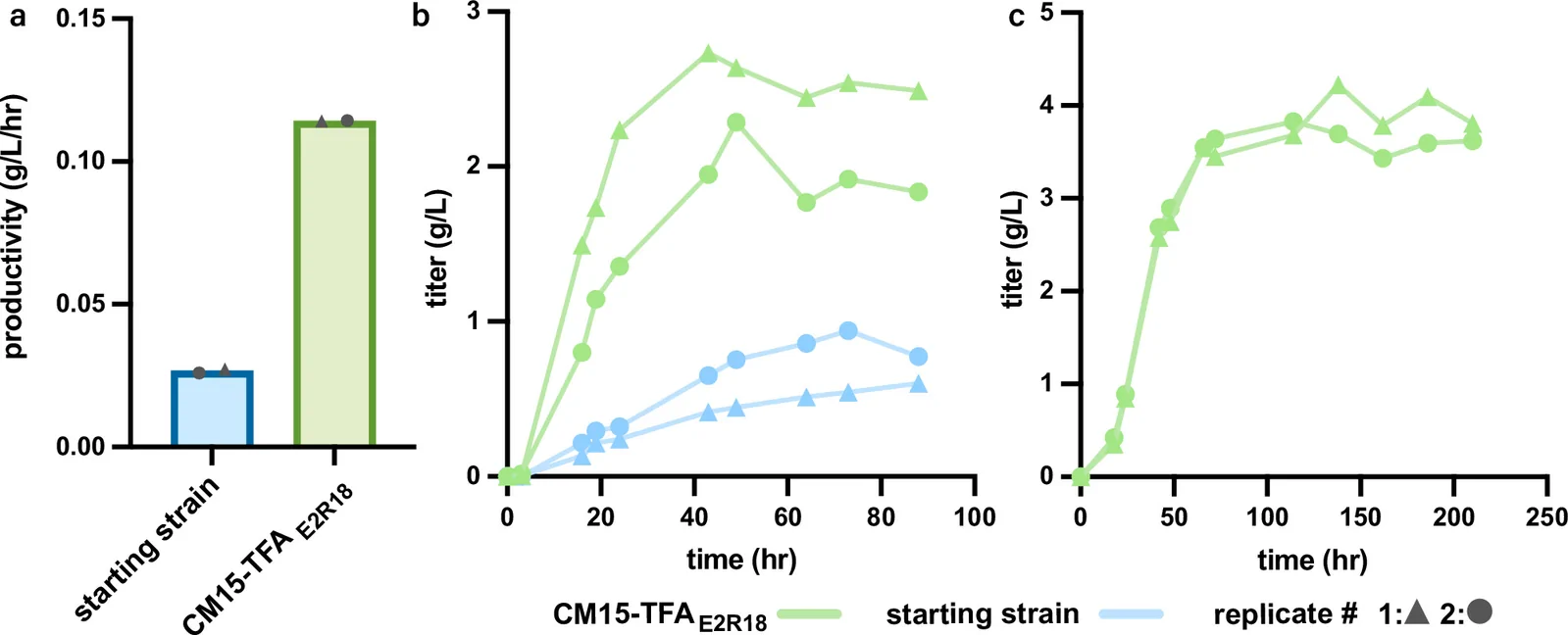
Anaerobic, fed-batch fermentation of starting CM15-TFA and evolved CM15-TFA_E2R18_ strains. **a** Maximum productivity of each strain in anaerobic, fed-batch conditions. CM15-TFA_E2R18_ achieved a maximum productivity that was much higher than the starting strain (CM15-TFA). Data represent the mean of biological replicates (*n* = 2). **b** Combined titer of all fatty alcohols produced over the course of each fermentation showing an approximately threefold increase in maximum titer achieved for the evolved CM15-TFA_E2R18_ strains. **c** Combined titer of all fatty alcohols produced in a fed-batch fermentation of CM15-TFA_E2R18_ using a 20% v/v dodecane overlay to mediate product toxicity. Source data are provided as a Source Data File.

Larger reaction volumes allowed us to sample our fermentations regularly and determine fatty-alcohol productivity over the course of the fermentation (Supplementary Fig. 1a). We hypothesized that, in a growth-coupled system, ALE-driven increases in growth rate would translate into higher fatty-alcohol productivity in the evolved strain CM15-TFA_E2R18_. Indeed, maximum productivity in the evolved CM15-TFA_E2R18_ was greatly enhanced at 110 ± 0.15 mg/L/hr relative to the starting strain’s productivity of 26.6 ± 0.95 mg/L/hr (Fig. 3a).

In fed-batch cultivation CM15-TFA_E2R18_ was able to reach a higher maximum titer than CM15-TFA. CM15-TFA_E2R18_ fermentations reached 2.5 ± 0.28 g/L of total fatty alcohols compared to 0.77 ± 0.24 g/L for the starting CM15-TFA strain (Fig. 3b). The product profile for each fermentation was roughly comparable with each strain producing similar relative amounts of each chain length (Supplementary Fig. 1b). In fed-batch fermentation, where culture conditions could be better controlled, the cell density achieved was much higher for the CM15-TFA_E2R18_ strain (Supplementary Fig. 1c). This increase in final OD_600_ translated to an increase in the growth-linked production of fatty alcohols.

Glucose consumption was monitored to enable yield calculation (Supplementary Fig. 1d). The theoretical yield of fatty alcohols was predicted using flux balance analysis in a condition where cells maximized growth rate by rebalancing their redox state entirely through rBOX (Supplementary Fig. 1e). The percentage of theoretical yield at the point each replicate achieved its maximum titer for the CM15-TFA_E2R18_ fermentations was 62% and 89% for replicates 1 and 2, respectively, compared to 67% and 42% for replicates 1 and 2 of the CM15-TFA fermentations.

The steep decline in optical density observed (Supplementary Fig. 1c) immediately after the fatty alcohol titer reached its maximum around 40 h (Fig. 3b) indicated potential product toxicity issues. We hypothesized that accumulation of fatty alcohols may lead to a toxic condition untenable for further growth^65^. To investigate this, we also cultivated CM15-TFA_E2R18_ with a 20% (v/v) dodecane overlay that could help partition fatty alcohols out of the aqueous culture and mitigate product toxicity^66^. This intervention was successful in raising the maximum titer of total fatty alcohols produced in each bioreactor to 4.0 ± 0.28 g/L (Fig. 3c). The observed maximum productivity was approximately the same as that observed in the experiment without overlay (0.097 ± 0.002 g/L/hr) (Supplementary Fig. 1f). Maximum optical density (Supplementary Fig. 1g) and glucose consumed (Supplementary Fig. 1h) were also improved in the overlay condition.

Overall, these results demonstrate that the adaptive evolution of the strain was effective in improving not only the growth kinetics but also the production metrics, demonstrating ALE as an important tool for untargeted engineering of industrial bioproduction hosts.

### Techno-economic analysis highlights the economic importance of an anaerobic bioprocess using a minimal medium

To contextualize the cost implications of implementing reverse β-oxidation anaerobically in a minimal medium, we performed a techno-economic analysis. We simulated bioprocesses using rich and minimal media under anaerobic conditions. We compared these processes with an aerobic process using both media, assuming the same titer, rate, and yield. We first analyzed the economics of bioproduction at our current state of technology using the observed titer, rate, and yield (Supplementary Data 1). The model predicted a 53% decrease in minimum selling price (MSP) for an anaerobic fatty-alcohol bioprocess using minimal medium compared with an aerobic process using the same medium. The use of rich medium increased the MSP (at the current state of technology) by approximately 35% for the anaerobic process and 17% for the aerobic process (Supplementary Fig. 2a). Differences in bioconversion cost dominated the changes in MSP across scenarios, with an anaerobic process in minimal medium costing approximately one fifth of that of an aerobic process using rich medium (Supplementary Fig. 2b). Capital expenditure (CapEx) for an anaerobic bioconversion section was also approximately one half of that for an aerobic counterpart (Supplementary Fig. 2c). Results from the current state-of-technology analysis were consistent with a simulated future fatty-alcohol bioprocess which operates near the efficiency of commercial ethanol fermentation (Supplementary Data 1), which likewise showed marked decreases in bioconversion cost for an anaerobic process using minimal medium (Supplementary Fig. 2d–f). At the current state of technology, the aerobic process requires a sixfold more capital-intensive onsite energy generation unit compared to the anaerobic process, and even after full optimization, it remains twice as capital-intensive as the anaerobic process. Both capital expenditures and energy requirements for the bioconversion process increased more steeply with increasing retention time in the aerobic process than in the anaerobic process. Biogas generated from unutilized sugars partly offsets the plant energy demand, reaching ~44% (anaerobic) and ~4% (aerobic) in the SOT case due to unutilized xylose, and ~21% and ~7%, respectively, under optimized conditions, thereby reducing purchased energy use and operating costs (Supplementary Fig. 3a). Collectively, these results underscore the advantages of the anaerobic configuration, as it offers greater operational flexibility, even when accounting for the extended bioconversion times required to achieve higher titers. Sensitivity analysis shows that the sugar-to-fatty alcohol yield and sugar feedstock cost are the most influential parameters affecting total production cost across all process configurations. Additionally, for the aerobic process, bioconversion time and aeration rate are the next most influential parameters after yield (Supplementary Fig. 3b,c). Together, these analyses underscore the importance of demonstrating our reverse β-oxidation platform under anaerobic conditions in minimal medium as a step toward economic viability, particularly in commodity markets where margins are thin and bioprocess costs can determine feasibility.

### Genetic characterization of evolved strains

**Fig. 4:**
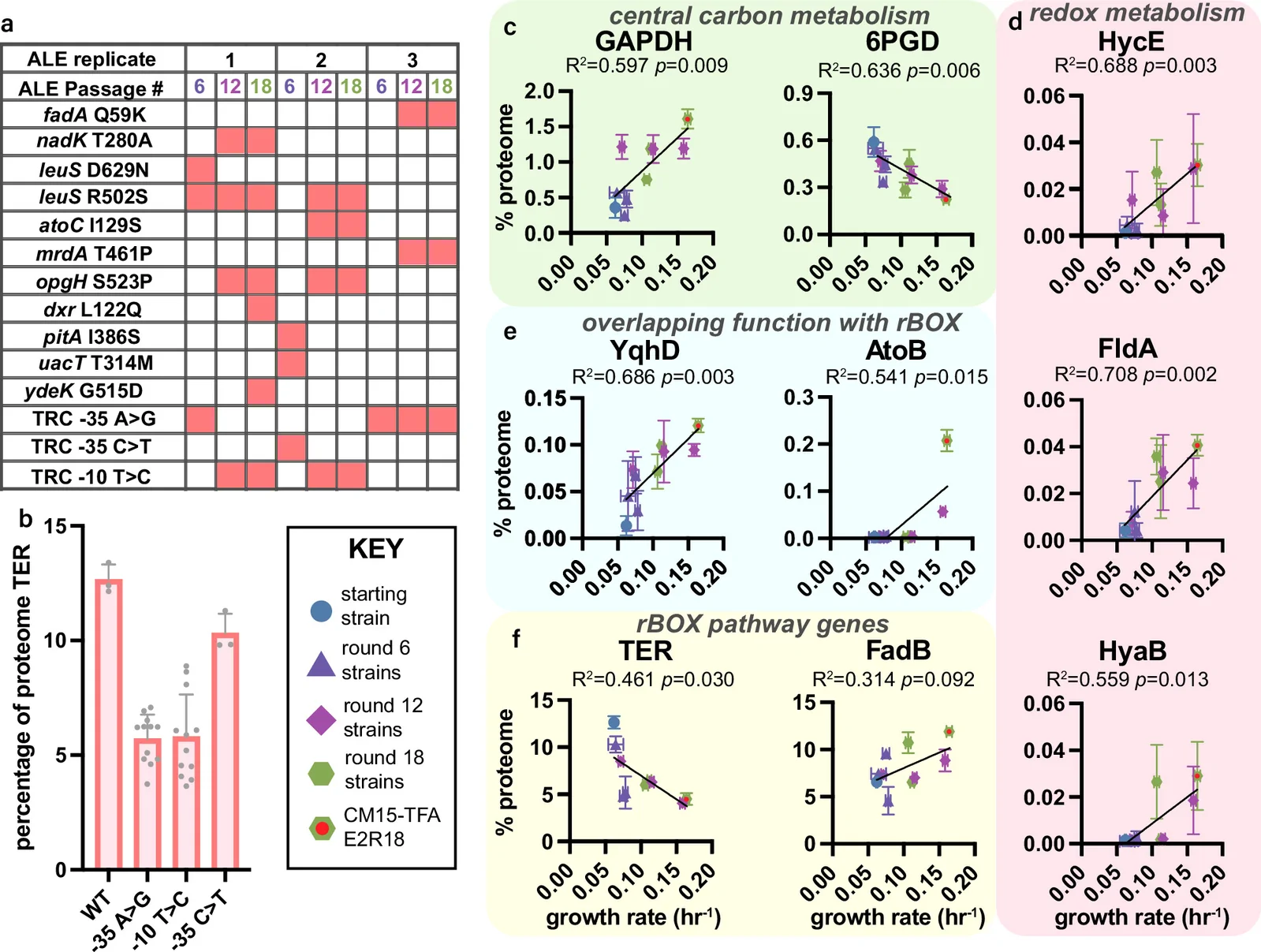
Genetic and proteomic characterization of evolved strains. **a** Table showing the mutations acquired over the course of the ALE. Red-filled squares in the table indicate the presence of the corresponding mutation in a given isolate. **b** TER expression in isolates with different mutations to the upstream TRC promoter and WT TRC promoter showing decreased expression of TER by the various TRC mutants. (WT: *n* = 3, −35 A > G: *n* = 12, −10 T > C: *n* = 10, −35 C > T: *n* = 3). **c** Correlation analysis between the expression levels of key central metabolic enzymes (GAPDH and 6PGD) and the average initial growth rate (*n* = 3). **d** Correlation analysis between the expression levels of redox homeostasis and hydrogen metabolism enzymes (HycE, FldA, and HyaB) and the average initial growth rate (*n* = 3). **e** Correlation analysis between the expression levels of enzymes whose function overlaps with that of the overexpressed rBOX pathway genes (YqhD and AtoB) and the average initial growth rate (*n* = 3). **f** Correlation analysis between the expression levels of overexpressed rBOX pathway genes (TER and FadB) and the average initial growth rate (*n* = 3). For **c**–**f**, relationships were assessed by simple linear regression accounting for the number and SD of biological replicates. *p* values were obtained from F tests of the null hypothesis that the slope equals zero, equivalent to a two-sided test, without adjustment for multiple comparisons. Exact *p* and R² values are shown in each panel. Data represent the mean ± s.d. of biological replicates. Source data are provided as a Source Data File.

To better understand the determinants of the observed increases in growth rate, productivity, and titer, we sequenced the genome of CM15-TFA_E2R18_ as well as the fastest-growing isolates from ALE replicates at rounds 6, 12, and 18. We identified 10 unique genomic mutations in the 9 sequenced strains as well as 4 unique mutations in the plasmid DNA expressing the rBOX biosynthetic genes (Fig. 4a).

Early in the course of the ALE each strain acquired a mutation in either the −35 or −10 element of the TRC promoter responsible for expression of the TER and ACR genes (Supplementary Fig. 4). These mutations may interfere with the recognition of the TRC promoter by RNA polymerase holoenzyme, which binds specifically to these elements through DNA interactions with sigma factor. Strains containing the TRC mutations fixed in later rounds (−35 A > G and −10 T > C) show a roughly twofold reduction in downstream TER expression (Fig. 4b), indicating that these mutations have an effect on expression tuning of this key pathway gene.

The rBOX pathway gene *fadA* acquired a Q59K substitution, which is located at the homodimer interface (Supplementary Fig. 5a). However, this substitution did not have an effect on protein expression (Supplementary Fig. 5b) or enzyme kinetics (Supplementary Fig. 5c). The gene *nadK* was also mutated, resulting in a T280A substitution (Supplementary Fig. 5d). NadK is responsible for the phosphorylation of NAD^+^ to yield NADP^+^. This mutation could have implications for redox homeostasis. However, expression of this protein was not detected by proteomic analysis in any of the samples.

Mutations were also found in genes that regulate cell envelope biosynthesis and function, including *mrdA* and *opgH*. *opgH* encodes an inner membrane glucosyltransferase that contributes to osmoregulated periplasmic glucan biosynthesis, osmotic adaptation, and regulation of cell division^67–69^. *mrdA* catalyzes crosslinking of the peptidoglycan cell wall^70^ and is responsible for the maintenance of the rod-shape of *E. coli*^71^. Mutations in these proteins may allow the cell to better tolerate the membrane or cell wall stress induced by production of fatty alcohols. To test this hypothesis, we used Multiplex Automated Genome Engineering (MAGE) to introduce the *opgH* S523P mutation into *E. coli* MG1655, generating the strain MG1655 *opgH* S523P. We then assayed the growth of this strain in a medium containing either no fatty alcohols or 0.3 g/L octanol to impose fatty alcohol stress. In the absence of fatty alcohol stress, MG1655 *opgH* S523P behaved similarly to the WT strain (Supplementary Fig. 5e). When exposed to 0.3 g/L octanol, MG1655 *opgH* S523P exhibited a modest, but significant shortening of the lag phase compared to the WT strain, consistent with the *opgH* S523P mutation promoting tolerance to fatty alcohols (Supplementary Fig. 5f, g).

Mutations were also present in regulatory genes or other genes associated with transcription/translation. An I129S mutation was present in the regulatory gene *atoC* which encodes a DNA-binding transcriptional activator responsible for regulating the *atoDAEB* operon. This operon encodes enzymes and transporters that mediate the reversible catabolism of short-chain fatty acids. Crucially, this operon encodes AtoB, an acetyl-CoA thiolase with overlapping function to FadA. In the two strains containing the *atoC* mutation, the *atoDAEB* operon, and importantly the AtoB protein, were upregulated (Supplementary Fig. 6). Notably, this exact mutation has been previously described as conferring constitutive activator activity to AtoC^72^, and this mutation has been rationally engineered into *E. coli* for the purpose of improving rBOX activity for shorter substrates^57^. This highlights the power of our non-targeted, ALE-based strategy to uncover strain enhancements that might otherwise demand extensive prior knowledge and laborious engineering. In addition to *atoC*, mutations were also observed at two positions within the leucyl-tRNA synthetase gene *leuS*. While LeuS is not a direct regulator of gene expression, perturbations to tRNA synthetase function have been shown to alter gene expression and proteome allocation more globally^73,74^.

### Proteomic characterization of evolved strains

To assess perturbations to gene expression that may not be obvious from sequenced mutations but that impacted growth rate, correlation analysis was performed. We determined the statistical significance of the correlation between growth rate and protein expression for each protein quantified in the proteome. We identified a number of proteins with statistically significant correlations. We categorized a subset of the identified proteins for which expression correlated to growth rate into four distinct groups: central carbon metabolism, redox metabolism, overlapping functions with rBOX pathway proteins, and rBOX pathway proteins (Fig. 4c–f).

We found the expression of two important proteins in central carbon metabolism to be altered in strains with different growth rates. Glyceraldehyde-3-phosphate dehydrogenase (GAPDH) was upregulated and 6-phosphogluconate dehydrogenase (6PGD) was downregulated in strains with faster growth rates (Fig. 4c). These enzymes each catalyze key reactions in divergent pathways for central carbon flux. 6PGD catalyzes the first committed step of the pentose phosphate pathway, a decarboxylative pathway responsible for generating NADPH whereas GAPDH catalyzes the key NADH-generating reaction in glycolysis. Together, these alterations to gene expression may shift metabolic pathways away from NADPH-generating reactions and increase the flux of a reaction generating NADH, the critical cofactor for rBOX that provides the driving force for the reversal of β‑oxidation. In agreement with this hypothesis, the intracellular concentration of pentose phosphate pathway intermediates was decreased in the evolved CM15-TFA_E2R18_ strain relative to CM15-TFA (Supplementary Fig. 7a).

Other proteins related to redox metabolism showed altered expression in evolved clones. The large subunit of formate hydrogen lyase (FHL), HycE, was upregulated in later rounds and its expression was significantly correlated with growth rate (Fig. 4d). Under anaerobic conditions, flux from glucose to acetyl-CoA is shunted through pyruvate formate lyase (PFL), leading to the accumulation of toxic levels of formate. To detoxify the formate produced by PFL, *E. coli* employs the activity of FHL to convert the formate to non-toxic hydrogen. Upregulation of FHL may help detoxify formate accumulated by increased flux to acetyl-CoA when the cell was engineered to use rBOX as the primary fermentation pathway. In CM15-TFA_E2R18_ intracellular concentrations of pyruvate were lower than in the starting strain and acetyl-CoA was higher, indicating a debottlenecking of metabolism at this metabolic branch point (Supplementary Fig. 7b). Flavodoxin (FldA), a redox carrier which supports the activity of PFL, among other redox active proteins, was also correlated with growth rate and upregulated in later rounds, providing another possible explanation for increased flux through PFL (Fig. 4d)^75^. Interestingly, the expression of the large subunit of the membrane-bound hydrogenase-1 (HyaB) was also correlated with growth rate, further implicating alterations to hydrogen metabolism in the adaptation of these strains to the use of rBOX as the primary fermentation pathway (Fig. 4d). To confirm alterations in the ability of starting and evolved strains to produce hydrogen we assayed the amount of hydrogen present in the headspace of our sealed culture vials after a 3-day fermentation (Supplementary Fig. 8a). We found an increase in hydrogen production in strain CM15-TFA_E2R18_ and were also able to show significantly increased expression of other hydrogenase maturation factors in the evolved strain (Supplementary Fig. 8b).

Enzymes with potential to participate in core rBOX reactions also showed significant differences in expression as a function of growth rate and were upregulated over the course of the ALE. Endogenous enzymes, namely YqhD and AtoB, which perform reactions overlapping those in rBOX, were upregulated over the course of the ALE (Fig. 4e). The protein YqhD is a NADPH-dependent aldehyde reductase that converts a broad range of aldehydes to their corresponding alcohols, contributing to detoxification and redox balancing in *E. coli*^76^. This enzyme’s function partially overlaps with that of ACR and could serve to detoxify fatty aldehydes generated by partial reduction of fatty acyl-CoAs by ACR. The protein AtoB is a thiolase analogous to FadA, but specific for short-chain acyl-CoAs with high activity for acetyl-CoA as a substrate^77^. AtoB has been shown to support the activity of rBOX by catalyzing the initial condensation of two acetyl-CoAs for which FadA has limited activity^61^.

Finally, the core rBOX genes overexpressed in CM15-TFA were altered over the course of the ALE, and the changes in their expression were correlated with growth rate (Fig. 4f). As mentioned previously, the enzyme TER was downregulated through mutations to its promoter (Fig. 4b). These changes to expression were significantly correlated with the growth rate of the evolved clones (Fig. 4f). The enzyme FadB, which catalyzes the reduction and dehydration of the 3-oxo rBOX intermediate (Fig. 1a), was upregulated over the course of the ALE with a nearly twofold increase in expression in the fastest-growing strain CM15-TFA_E2R18_. While the correlation between growth rate and expression did not cross our threshold for significance, this trend is notable as FadB has been previously identified as the rate-limiting step in our rBOX system^78^. These results suggest that, while protein expression from the initial construct was sufficient to enable growth of CM15, the selection scheme fine-tuned gene expression across the rBOX pathway, downregulating genes expressed above their optimal level and upregulating genes that may previously have limited flux. These results highlight a key advantage of this growth-coupled evolution technique, which appears to be able to fine-tune enzyme expression across the entire rBOX pathway to maximize flux, reducing the need for targeted intervention to circumvent pathway bottlenecks.

## Discussion

This study establishes rBOX as a synthetic anaerobic fermentation pathway capable of supporting the growth of fermentation-deficient *E. coli* by balancing its redox state under anaerobic conditions. By engineering a fermentation-deficient strain to rely upon rBOX for redox balancing, we created a unique growth-coupled phenotype that links cell proliferation directly to the biosynthesis of fatty alcohols. This coupling not only confirmed the thermodynamic and metabolic sufficiency of rBOX but also provided a strong selection pressure to drive strain improvement through ALE.

The implementation of ALE in this context proved highly effective and straightforward. In contrast, traditional metabolic engineering strategies to improve pathways like rBOX require massive investments in personnel and resources^14,79^. The complexity of engineered biological systems makes strain improvement knowledge-intensive; when required changes are beyond current understanding, progress stalls and demands system-specific, intensive study. However, using an untargeted, growth-coupled approach allowed us to make a greater than threefold improvement in final titer and a fourfold improvement in productivity without any direct manipulation of the strain. This untargeted and facile approach, if applied more generally, would reduce the time and cost associated with strain development, which are key constraints to realizing a sustainable bioeconomy^13,14^. Furthermore, our growth-coupled approach allowed us to realize a titer of fatty alcohols that had not previously been achieved under anaerobic conditions, demonstrating the ability to not only arrive at an engineering outcome faster and more easily using growth coupling, but also to push beyond the limits encountered in previous work which relied upon rational design.

Our untargeted, ALE-based approach resulted in modifications to the strain that would have been not only difficult to predict from a rational engineering standpoint, but also technically difficult to engineer. Not only were alterations to gene expression that route metabolism away from the pentose phosphate pathway difficult to predict, but achieving the optimal level of expression rationally would require laborious testing of a multitude of different expression systems. Even in the four key rBOX pathway genes, which are obvious targets for expression tuning, testing five different expression levels for each gene combinatorially would require assaying over 500 different constructs. By mimicking the random walk of natural evolution, our system was able to overcome the limitations of laborious rational engineering strategies and uncover unexpected adaptations that cumulatively allowed for the marked improvement of our bioproduction strain. More broadly, growth coupling combined with adaptive evolution could provide a straightforward and low-cost route to improving strains that produce diverse bioproducts. However, redox-balance-based growth coupling carries inherent stoichiometric constraints, which can limit its suitability for products that do not consume sufficient reducing equivalents to serve as effective redox sinks. Pathways whose redox and carbon stoichiometries differ from native fermentation pathways may therefore require additional design strategies^52^.

By scaling the evolved strains up into bioreactors, we were able to uncover and quantify performance gains that accumulated over the course of ALE. These results underscore the value of pairing metabolic engineering with process engineering to fully realize strain improvements. In our case, product toxicity was mitigated by using an organic overlay; future work could apply adaptive evolution specifically to further increase product tolerance of the strain itself. Additional gains may also be achievable through advanced bioprocess strategies such as in situ product recovery. Finally, because these strains are growth-coupled they gain, or at least maintain production capacity over time and are less sensitive to genetic drift, which causes many other bioproduction chassis to lose production capacity over the course of fermentation^16^. This feature makes them well suited to emerging and mature^3,80^ continuous fermentation approaches where bioproduction cost can be further reduced by decreasing waste and reactor downtime.

Techno-economic analysis reveals that the production of fatty alcohols under anaerobic conditions in minimal medium markedly improves process economics compared with aerobic and rich-medium alternatives. These results underscore the importance of developing bioproduction processes for price-sensitive commodities under anaerobic conditions in minimal medium. Our growth-coupled system which operated strictly under anaerobic conditions in minimal medium allowed the cells to evolve and adapt to the most economically viable production environment, enabling anaerobic fatty-alcohol production at a titer not previously achieved for our observed products^56,81,82^. The technological readiness of the production chassis described here could be further improved through genomic integration of the pathway enzymes. Provided that single-copy genomic integration enables sufficient expression of the rBOX pathway genes, this approach could enhance strain and production stability, reduce cell-to-cell variability, and eliminate the need for antibiotics, thereby decreasing process costs and improving industrial relevance. This work provides a roadmap for the continued development of bio-produced commodities to displace petrochemicals through the development of synthetic anaerobic fermentation.

Overall, this manuscript details a workflow for strain development that circumvents the traditional, laborious methods of strain development used to engineer industrial hosts. By recognizing the bioenergetic requirements of rBOX as complementary to the needs of *E. coli* in the absence of canonical fermentation, we were able to generate a robust and stable selection system allowing for long-term growth-coupled evolution. This work demonstrates the utility of complementation of a synthetic deficit to metabolism by an engineered bioproduction pathway when coupled with ALE. By extending this strategy to other growth-coupled pathways, this framework could be broadly applied to accelerate and enhance metabolic engineering efforts and drive the development of a sustainable bioeconomy.

## Methods

### Growth medium

Bacterial strains were routinely cultured and propagated in 2xYT medium (BD Difco™), supplemented with the appropriate antibiotic based on the plasmid(s) present in the strain: carbenicillin (100 µg/mL), kanamycin (50 µg/mL), or apramycin (50 µg/mL). Growth and/or production experiments were conducted in a minimal M9 medium containing 1× M9 Salts (Sigma), supplemented with 2 mM MgSO_4_, 0.1 mM CaCl_2_, 4.98 mg/L FeCl_3_, 0.84 mg/L ZnCl_2_, 0.765 μM CuCl_2_, 0.420 μM CoCl_2_, 1.60 μM H_3_BO_3_, and 0.081 μM MnCl_2_.

### Plasmid assembly

Plasmid pBbA6a_fadAB was assembled using HiFi assembly (NEBuilder). The plasmid backbone used for assembly was amplified from plasmid pBbA6a_RFP^83^ using forward primer BC1 and reverse primer BC2. The *fadAB* operon was amplified from *E. coli* MG1655 gDNA using forward primer BC3 and reverse primer BC4. The PCR reactions were performed in 50 μL reactions according to the Q5 polymerase protocol. The assembled plasmids were sequence-verified (Plasmidsaurus).

Plasmid pSeva_TER_ACR was also assembled through HiFi assembly. The plasmid backbone used for assembly was amplified from pSeva^84^ using forward primer BC5 and reverse primer BC6. The trans-2-enoyl-CoA reductase gene from *Treponema denticola* was codon optimized for *E. coli* and synthesized as a double-stranded DNA fragment (Genscript). Acyl-CoA reductase (ACR) from *Marinobacter aquaeolei* VT8 was amplified from plasmid pDVA02085 (Registry #JPUB_010419)^85^ using forward primer BC7 and reverse primer BC8. Once both parts and the backbone were purified they were assembled according to the HiFi-Assembly protocol, transformed, and sequence verified.

All primers were synthesized by Integrated DNA Technologies (IDT).

### Small scale fermentation

Overnight cultures (5 mL) grown at 37 °C in 2xYT were washed 3 times with M9 medium before inoculation at a 20-fold dilution into an M9 medium containing 2% glucose and appropriate antibiotics for preculture. The preculture was conducted aerobically at 37 °C with shaking at 200 rpm. Once OD_600_ reached 0.4–0.6, about 2 h after inoculation, cultures were induced with isopropyl β-D-1-thiogalactopyranoside (IPTG) to a final concentration of 1 mM and further cultivated for 3 h to express rBOX proteins. 18 × 150 mm borosilicate glass culture tubes (Chemglass) were prepared with 5 mL of M9, 2% glucose, appropriate antibiotics and 1 mM IPTG. Three hours post induction the preculture was diluted into the prepared borosilicate glass culture tubes at a starting OD_600_ of 0.05. Culture tubes were sealed with blue chlorobutyl stoppers (Chemglass) and aluminum seals (Agilent) and the headspace of the sealed culture tubes was flushed with nitrogen for 2 min to remove oxygen. Once the tubes were purged of oxygen they were held at an angle of 45 degrees at 37 °C with 200 rpm shaking for fermentation. Average initial growth rate (hr^−1^) was calculated based on the change in growth per unit time between inoculation and the first OD_600_ measurement 17 h post inoculation.

### Adaptive laboratory evolution and whole genome sequencing

Three cultures of CM15-TFA containing the rBOX pathway were prepared for small scale fermentation as described above. Each culture was then iteratively passaged to generate distinct ALE lineages. Once each culture had grown for 5 days anaerobically in minimal M9 medium it was rescued by diluting the culture 1:50 into 2xYT and growing overnight. The rescued cells were then used for the next round of each ALE lineage. This evolution took place for 18 rounds.

Single colonies from the evolving culture were isolated to determine the genetic mutations associated with improved growth performance. Plasmids from these isolates were sequenced using Oxford Nanopore technology (Plasmidsaurus). Whole genome sequencing was performed using short-read Illumina technology (Plasmidsaurus).

For whole genome sequencing, raw reads were quality-filtered and trimmed using AfterQC (v0.9.7)^86^. The processed reads were mapped to the *E. coli* MG1655 reference genome (U00096.3) using the breseq pipeline (v0.33.1)^87^. The consensus frequency threshold was set to 0.75, with a required match fraction of 0.95. The breseq analysis was executed in clonal mode. Mutation identification and annotation were carried out using the ALEdb workflow^88^. Sequencing coverage averaged over 300× per base across analyzed genomes.

### Fed-batch bioreactor fermentation

Overnight cultures of the starting CM15-TFA and evolved CM15-TFA_E2R18_ strains were grown in 2xYT at 37 °C with shaking at 200 rpm. The cultures were washed three times with 1× M9 salts before being inoculated into 50 mL M9 containing 2% glucose and appropriate antibiotics in shake flasks at a 20-fold dilution. The flasks were grown at 37 °C with shaking at 200 rpm until OD_600_ reached 0.4–0.6 at which time they were induced with 1 mM IPTG. Three hours post induction they were inoculated into Eppendorf DASGIP 1.5 L bioreactors in duplicate.

Fed-batch fermentations were performed in a four-vessel Eppendorf DASGIP bioreactor system. When two strains or conditions were compared in parallel within the same run, the system capacity limited the number of biological replicates to *n* = 2 per group. Thus, productivity measurements in Fig. 3 were obtained from duplicate bioreactor vessels for each strain/condition.

Bioreactors were initially charged with 500 mL M9 containing 2% glucose, antibiotics, and 1 mM IPTG with temperature set to 37 °C and agitation rate set to 400 rpm. An initial nitrogen purge using an L sparger was done to remove any dissolved oxygen as well as headspace oxygen. A constant 0.06 L/h flow rate of nitrogen was maintained during the course of the experiment to prevent any ambient oxygen from dissolving into the reactors. The pH setpoint was maintained at 7.0 ± 0.1 using 15% (w/v) NH_4_OH. Approximately every 24 h, a 50 mL bolus of 1× M9, 25 g/L glucose, antibiotics, and 1 mM IPTG was added to the reactors to account for liquid level loss due to sampling as well as for feeding additional glucose.

The CM15-TFA_E2R18_ inoculum for fed-batch fermentation with a 20% (v/v) dodecane overlay was prepared in the same way as fermentation without overlay. Bioreactors were initially charged with 500 mL M9 containing 20 g/L glucose, antibiotics, and 1 mM IPTG with temperature set to 37 °C and agitation rate set to 400 rpm. A 20% (v/v) dodecane overlay was added prior to inoculation. An initial nitrogen purge using an L sparger was done to remove any dissolved oxygen as well as headspace oxygen. A consistent 0.06 L/hr flow rate of nitrogen was maintained during the course of the experiment to prevent any ambient oxygen from dissolving into the reactors. The pH setpoint was maintained at 7.0 ± 0.1 using 15% (w/v) NH_4_OH. Reactors were fed with glucose at 24 and 48 h to reach 20 g/L for continued bioproduction.

### Multiplex automated genome engineering (MAGE)

*E. coli* strain MG1655 harboring plasmid pORTMAGE-2 was cultured at 30 °C in 2xYT overnight before inoculation at a 100-fold dilution into fresh 2xYT media for preculture at 30 °C. Once OD_600_ reached 0.2, cells were induced by thermal shock at 42 °C using a warm bath for 13 min to induce expression of CspRecT recombinase and EcMutL-E32K mismatch repair repressor proteins. Cells were then immediately chilled on ice for 1 h. After chilling, the cells were made electrocompetent by washing three times with ice-cold 10% glycerol. Finally, the cells were suspended in 50 μL of 10% glycerol and 1 μL of 100 μM *opgH* MAGE primer BC9 was added. The cells were electroporated at 1.8 kV and rescued by addition of 1 mL 2xYT and grown at 37 °C for 3 h. Post-rescue, cells were plated onto LB agar plates and grown at 37 °C. Colonies were assayed for incorporation of the desired mutation by colony PCR and verified by Sanger sequencing.

### Fatty alcohol tolerance of *E. coli* WT MG1655 and MG1655 *opgH* S523P

Overnight cultures were diluted 20-fold into fresh 2xYT medium and grown at 37 °C with shaking at 200 rpm. Once cultures reached an OD_600_ of approximately 1.0, cells were diluted into M9 medium containing 2% glucose in a 96-well plate to a starting OD_600_ of 0.05. Each well contained 150 μL of culture. To assess fatty alcohol tolerance, octanol was added to the indicated wells at a final concentration of 0.3 g/L. Plates were sealed with a Breathe-Easy® sealing membrane and incubated at 37 °C with shaking in a FilterMax M2 spectrophotometer, with OD_600_ monitored over time.

### Flux balance analysis

COBRApy was employed for flux balance analysis with modifications made to the iJO1366 metabolic model for *Escherichia coli*^89,90^. The metabolic model was modified with genetic knockouts to reflect the genotype of strain CM15 following a similar rationale to Mehrer et al. Additional knockouts were made to simulate strain metabolism under anaerobic conditions (for example, pyruvate dehydrogenase was knocked out to shuttle pyruvate flux to pyruvate formate lyase). To create the rBOX pathway we added reactions for each extension of acetyl-CoA and subsequent enzymatic reactions (Fig. 1a). More details and access to the Jupyter Notebook used in this analysis are available at https://github.com/lbincha/Modeling_2025.

### Techno-economic analysis

We performed a techno-economic analysis to estimate the minimum selling price (MSP) of fatty alcohols produced via an anaerobic bioprocess and to compare (i) anaerobic versus a representative aerobic process and (ii) minimal (M9) versus rich (LB + MOPS) laboratory media under matched fermentation performance (same titer, rate, and yield for the aerobic/anaerobic comparison). A commercial-scale facility was modeled using the chemical engineering process simulation software SuperPro Designer^91^, processing 1,000 bone-dry metric tons (bdt) per day of lignocellulosic sugar. Two scenarios were evaluated: a current state-of-technology (SOT) case with 20% (v/v) dodecane overlay and an optimal future case without dodecane overlay, assuming that the process can be engineered to operate efficiently without an external organic phase. Although a maximum titer of 4.0 ± 0.28 g/L was achieved at a retention time of 138 h (Fig. 3c), we selected an average titer of 3.75 g/L at a retention time of 114 h (Fig. 3c) for the SOT economic analysis, as the increase in titer with longer retention time did not substantially reduce fatty alcohol production cost. In the SOT case, conversion was modeled using glucose only to reflect experimental constraints, and xylose in the sugar stream was assumed unutilized and routed to wastewater treatment. In the future case, xylose utilization was assumed to reach 90% of the glucose conversion efficiency, and process improvements were assumed including reduced sugar cost and improved fermentation titer, rate, and yield, targeting 90% of the theoretical sugar-to-product conversion efficiency (Supplementary Data 1). The assumed 90% theoretical sugar-to-fatty-alcohol conversion yield is an upper-bound, literature-informed target. It is consistent with near-theoretical yields reported for optimized microbial ethanol-production systems^92,93^, and is intended to represent a forward-looking scenario rather than current industrial performance. Unutilized sugars in both scenarios were sent to wastewater treatment with anaerobic digestion to produce biogas, which was combusted onsite to provide process heat and electricity. Downstream processing was modeled assuming centrifugation for cell removal, followed by decantation and distillation to recover a mixed fatty alcohol stream suitable for use as a diesel blendstock; no further purification to isolate individual alcohols was included. Detailed separation assumptions, recovery efficiencies, and associated costs are provided in the accompanying Supplementary JSON flowsheet and deposited in the publicly accessible Project PISCES database (https://projectpisces.org/flowsheets/103).

Because defined and rich laboratory media are economically impractical for full-scale production reactors, M9 and LB + MOPS were assumed to be used only in seed reactors for inoculum preparation; the production reactor received the inoculum and was supplemented with low-cost nitrogen sources (corn steep liquor and diammonium phosphate). Material and energy balances were used to estimate equipment sizes, followed by capital and operating cost estimation. Equipment costs were adopted from prior studies^94–96^, scaled to the modeled capacity, and escalated to 2020 USD using a plant cost index to calculate total capital cost. Annual operating costs included biomass feedstock, process chemicals, waste disposal, utilities, and fixed costs (labor, maintenance, taxes, and insurance). The product stream was modeled as a mixture of fatty alcohols using the molar fractions specified in Supplementary Fig. 1b. MSP was calculated by discounted cash flow analysis with coproduct revenues credited at current market prices, assuming a 10% after-tax internal rate of return, 30-year plant life, 7,920 operating hours per year, and a 21% income tax rate^94,95^. Model input parameters are summarized in Supplementary Data 1. Additionally, a downloadable JSON file containing all unit process and flow data in a standardized flowsheet format is provided as part of the Supplementary Information and deposited in the publicly accessible Project PISCES database (https://projectpisces.org/?page=database).

### Proteomic analysis

Upon completion of fermentation, cultures were centrifuged at 5000 ×  *g* for 3 min to pellet cells. Protein was isolated from cell pellets, and tryptic peptides were generated following a standard protocol^40,97^. In brief, pellets were lysed in Qiagen P2 Lysis Buffer, proteins were precipitated with NaCl and acetone, and the resulting pellet was washed, then resuspended in ammonium bicarbonate/methanol. Protein concentration was determined using the DC protein assay (BioRad). Samples were reduced with tris(2-carboxyethyl)phosphine, alkylated with iodoacetamide, and digested overnight with trypsin at a 1:50 enzyme-to-substrate ratio.

Peptides were analyzed on an Agilent 1290 UHPLC coupled to a Thermo Scientific Orbitrap Exploris 480 mass spectrometer, using a C18 column and a 10-minute gradient between solvent A (0.1% FA in H_2_O) and solvent B (0.1% FA in ACN). Data-independent acquisition (DIA) was employed with 3 survey scans and 45 MS2 scans, spanning *m*/*z* 380–985^98^.

DIA raw data files were processed with DIA-NN in library-free mode using an *E. coli* UniProt proteome database together with heterologous protein sequences^99^. Automated mass-tolerance and retention-time optimization were enabled, and protein quantification used the Top3 approach^100,101^. DIA-NN outputs were filtered at a global FDR of 0.01 at both precursor and protein-group levels. Data have been deposited to ProteomeXchange^102^. DIA-NN is available at https://github.com/vdemichev/DiaNN.

### Structural prediction

Structures were predicted using the Boltz-2x structure prediction algorithm with default settings^103^ on the Foldy platform^104^. The multiple sequence alignment was generated using the mmseqs2 server. The Boltz family of models, originally based on the AlphaFold3 architecture, have been shown to have structure prediction accuracy comparable to crystallographic structures.

### Gas chromatography and mass spectrometry analysis of fatty alcohols

Samples (1 mL) were taken from fermentation vessels for quantification of products. Fatty alcohols were extracted at a 1:1 ratio of culture to hexane containing an internal standard (50 mg/L pentadecanol) and vortexed at 3000 rpm for 15 min. The tubes were centrifuged at 10,000 × *g* for 10 min to pellet all biomass and fully separate organic and aqueous phases. The organic phase was analyzed by gas chromatography–mass spectrometry (GC-MS).

GC-MS analysis was performed using an Agilent Technologies Intuvo 9000 GC System and 5977B MSD equipped with a 15 m × 0.25 mm, 0.25 μm Agilent DB-WAX UI column. A 1 μL volume of extracted fatty alcohols in hexane was injected. The temperature program was as follows: a hold time of 1 min at 50 °C, ramp to 250 °C at 20 °C/min, hold at 250 °C for 3 min. Spectra (40–550 *m*/*z*) were acquired at 2.9 scans/sec. Signal intensity was compared to an authentic standard to determine production titer, and was normalized to the signal intensity of the pentadecanol internal standard. Mass spectra were compared to authenticated standards.

Samples from fermentations containing 20% (v/v) dodecane overlays had the organic phase isolated and stored at −80 °C before analysis. The organic overlay samples were then diluted at a ratio of 1:100 overlay to hexane containing an internal standard (50 mg/L pentadecanol) and vortexed at 3000 rpm for 15 min. The tubes were centrifuged at 10,000 × *g* for 10 min to pellet any biomass. This organic phase was analyzed by gas chromatography with flame ionization detection (GC-FID).

GC-FID analysis was performed using an Agilent Technologies 8890 equipped with a 15 m × 0.25 mm, 0.25 μm Agilent DB-WAX UI column. The acquisition method included an inlet temperature of 250 °C, detector temperature of 300 °C, and 2.2 mL/min helium carrier-gas flow. A 1 μL volume of extracted fatty alcohols in hexane-overlay mix was injected. The temperature program was as follows: initial 1-min hold at 40 °C, a ramp of 25 °C/min to 200 °C, a 3 °C/min ramp rate to 230 °C, and a final 2.6-min hold at 230 °C. Signal intensity was normalized to pentadecanol internal standard analyte peak area. Normalized signals were compared to authenticated standards using the same acquisition method.

### High performance liquid chromatography for quantification of glucose

For quantification of glucose, high performance liquid chromatography with refractive index detection (HPLC-RID) was employed. Samples were prepared by centrifuging 250 μL of culture at 10,000 × *g* for 10 min and filtering the resulting supernatant through 0.2 μm filters into sample vials. An Agilent Series 1200 was used to deliver a 10 μL injection volume of clarified culture supernatant to an Aminex HPX-87H column (BioRad) at 65 °C with subsequent detection of analytes by refractive index detector (RID) maintained at 44 °C. This separation and quantification was performed in an aqueous 4 mM H_2_SO_4_ solution flowing at a rate of 500 μL/min for 26 min per sample.

### Protein expression and purification

Overnight cultures of BL21 (DE3) *E. coli* strains harboring expression vectors for FadA WT and FadA Q59K were transferred into a 500 mL TB + kanamycin culture grown at 37 °C. Upon cells reaching OD_600_ ~ 0.4 cultures were placed on ice for 10 min prior to addition of IPTG to a concentration of 0.1 mM. Cultures were then grown overnight at 30 °C for overexpression of FadA proteins.

After 18 h of overexpression, the cells were harvested and sonicated in 30 mL of lysis buffer (50 mM Tris-HCl pH 7.4, 300 mM NaCl, 10% (v/v) glycerol, 5 mM imidazole). Post lysis, the cells were centrifuged at 5000 × *g* for 15 min at 4 °C. Clarified lysates were isolated for purification. 6 mL of Ni-NTA resin beads (Thermo) suspended in lysis buffer were added to resulting lysates and equilibrated by rotation at 4 °C for 1 h. Equilibrated lysates were added to gravity flow columns to remove lysis buffer from resin beads. Non-His-tagged proteins were removed from the resin bed with 6 mL of wash buffer (50 mM Tris-HCl pH 7.4, 300 mM NaCl, 40 mM imidazole) by washing 4 times. His-tagged proteins were removed from Ni-NTA beads with elution buffer (50 mM Tris-HCl pH 7.4, 300 mM NaCl, 10% (v/v) glycerol, 200 mM imidazole) in 2 mL fractions. Purified fractions were buffer-exchanged to remove imidazole in 10 mL protein storage buffer (50 mM Tris-HCl pH 7.4, 300 mM NaCl, 10% (v/v) glycerol) using Amicon 30 K 50 mL centrifugal filters (Sigma) spun at 5000 × *g* for 15 min at 4 °C three times. Absorbance at 280 nm was measured from purified proteins to determine concentration.

### In vitro enzyme kinetics

FadA WT and FadA Q59K thiolase activity was determined in the degradative direction using acetoacetyl-CoA as a substrate. Standard assay conditions were 50 mM Tris-HCl, 10 mM MgCl_2_, 3 mM coenzyme A, pH 8.1 in a total volume of 15 μL. Acetoacetyl-CoA was added to the reaction mixture as a substrate in concentrations of 250-3000 μM. Reactions were initiated by the addition of 2 μg of enzyme. Measurement of the magnesium-enolate form of acetoacetyl-CoA degradation was monitored at 303 nm to determine kinetic parameters in a 384-well plate (Thermo Fisher Scientific) using a FilterMax M2 spectrophotometer at 37 °C.

### Metabolomic analysis

Metabolomic analysis was performed on lysates prepared from samples in mid-log growth phase. Cell cultures were pelleted at 14,000  × *g* at 4 °C for 2 min, immediately quenched with 250 μL ice cold MeOH, and mixed thoroughly. 250 μL of water was then added to the MeOH lysate and mixed thoroughly. The MeOH/water lysate was centrifuged at 14,000 × *g* at 4 °C for 10 min. 450 μL of the clarified lysate was transferred to Amicon 3 K 0.5 mL centrifugal filters (Sigma) and centrifuged at 14,000 × *g* at 4 °C for 60 min. 350 μL of the resulting filtrate was transferred to a 2 mL sample tube, followed by the addition of 600 μL water. This mixture was frozen with liquid nitrogen and lyophilized for 2 days, then reconstituted with 100 μL 1:1 MeOH and water to concentrate intracellular metabolites^105^.

LC–MS analyses were performed on a Waters ACQUITY UPLC BEH Amide column (100 × 2.1 mm, 1.7 µm) using an Agilent 1290 Infinity II UHPLC. Samples (1 µL) were kept at 4 °C and the column at 30 °C^106^. Mobile phase A was 10 mM ammonium acetate, 0.2% ammonium hydroxide, and 5 µM medronic acid in water; mobile phase B was the same buffer in 80% acetonitrile/20% water. The gradient was: 0–0.5 min, 100% B; 0.5–2.93 min, 100 → 75% B; 2.93–3.28 min, 75 → 70% B; 3.28–3.83 min, 70% B; 3.83–4.03 min, 70 → 50% B; 4.03–4.33 min, 50% B; 4.33–4.53 min, 50 → 100% B; 4.53–7.0 min, 100% B. The flow rate was 0.36 mL/min (0–4.33 min), ramped to 0.5 mL/min over 0.2 min, and held to 7.0 min. The UHPLC was coupled to an Agilent 6545 QTOF operated in negative ESI mode (AJS source, [M–H]–) with capillary 3500 V, drying gas 10 L/min at 300 °C, nebulizer 20 psi, sheath gas 12 L/min at 350 °C, nozzle 2000 V, fragmentor 100 V, skimmer 50 V, and OCT1 RF 300 V. Spectra (60–1100 *m*/*z*) were acquired at 0.86 spectra/s, and data were collected and processed with Agilent MassHunter software.

### H_2_ gas analysis

Gas analysis was conducted using a Shimadzu GC-2014 gas chromatograph with a thermal conductivity detector (TCD)^107^. 5 mL of the headspace of blue chlorobutyl-stoppered fermentation vials was sampled using a syringe and subsequently analyzed. Samples flowed isothermally through a 2 mm × 8 m Shimadzu column at 60 °C using argon as the carrier gas. H_2_ was quantified after 8.5 min of flow time through a TCD module at 120 °C. The gas profile was quantified relative to a 100% H_2_ injection.

### Statistical methods

Differences between two means were evaluated using an unpaired, two-tailed Student’s *t* test. Differences were considered statistically significant at *p* < 0.05.

To evaluate relationships between protein expression and cellular growth rate, simple linear regression was performed using GraphPad Prism version 10.2.1 (GraphPad Software, Boston, MA, USA). Mean relative protein abundance was regressed against mean growth rate for each strain, accounting for the number and SD of biological replicates. Both the slope and intercept were fitted. *P* values were calculated using F tests of the null hypothesis that the slope equals zero, equivalent to a two-sided test. No adjustment was made for multiple comparisons, and *p* < 0.05 was considered statistically significant.

### Reporting summary

Further information on research design is available in the Nature Portfolio Reporting Summary linked to this article.

## Supplementary information

Supplementary Information

Description of Additional Supplementary File

Supplementary Data 1

Reporting Summary

Transparent Peer Review file

## Source data

Source Data

## Data Availability

Source data, including annotated sequences of plasmids used in this study, are provided with this paper and are available for download at Figshare. Raw sequencing data have been deposited to the Genome Sequence Archive (GSA) with accession code CRA033850. Raw proteomic data have been deposited to the PRIDE database with accession code PXD070655. Raw metabolomic data have been deposited to the MassIVE database with accession code MSV000102903. Process flowsheet, equipment sizing and cost, and the standard JSON file of modeling data are available at [https://projectpisces.org/flowsheets/103]. Source data are provided with this paper.

## Code availability

All custom code used in this study is available at GitHub. Custom code used for metabolic modeling is available at: [https://github.com/lbincha/Modeling_2025]^108^. Code used for proteomic analysis is available at: https://github.com/vdemichev/DiaNN.
