# Supplementary material for: Replacing anaerobic fermentation with reverse β-oxidation enables growth-coupled evolution for enhanced fatty-alcohol production
Source: Nat Commun. 2026 Oct 14;17:11615. doi: 10.1038/s41467-026-78481-1 (PMC13754892; doi:10.1038/s41467-026-78481-1)
Supplement: Supplementary file 1 [file 41467_2026_78481_MOESM1_ESM.pdf]

Replacing anaerobic fermentation with reverse β-oxidation enables growth-coupled evolution for enhanced fatty-alcohol production

Bridgie Cawthon[1,2 †], Aidan E. Cowan[1 † *], Zen Yoshikawa[1,2], Kenneth Low[1], Justin Baerwald[1,2], Nawa Raj Baral[1], Matthew Incha[1,3], Jennifer Gin[1], Yan Chen[1], Christopher J. Petzold[1], Edward E. K. Baidoo[1], Emine A. Turumtay[1], Corinne D. Scown[1,4], Adam M. Feist[1,5,6], Jay D. Keasling[1,2,6,7,8 *]

[†]Denotes equal contribution, names ordered alphabetically

*Corresponding authors aidancowan@berkeley.edu, keasling@berkeley.edu

[1]Joint BioEnergy Institute, Lawrence Berkeley National Laboratory, Emeryville, CA 94608, USA
[2]Department of Chemical and Biomolecular Engineering, University of California, Berkeley, Berkeley, CA 94720, USA
[3]Department of Bioresource and Environmental Security, Sandia National Laboratories, Livermore, CA 94551, USA
[4]Energy & Biosciences Institute, University of California, Berkeley, Berkeley, CA 94720, USA
[5]Department of Bioengineering, University of California, San Diego, CA, USA
[6]The Novo Nordisk Foundation Center for Biosustainability, Technical University of Denmark, Kemitorvet, Building 220, Kongens Lyngby 2800, Denmark
[7]QB3 Institute, University of California, Berkeley, Berkeley, CA 94720, USA
[8]Department of Bioengineering, University of California, Berkeley, Berkeley, CA 94720, USA

Supplementary Table 1: Strains used in this study

Strain name	Genotype	Description	Registry #	Reference
MG1655	F⁻ λ⁻ ilvG⁻ rfb-50 rph-1	Wild-type *E. coli* K-12 strain used as the parental background for genome engineering.		[1]
CM15	*E. coli* MG1655 Δ*araBAD* Δ*fadRE* Δ*ldhA* Δ*ackApta* Δ*adhE* Δ*poxB* Δ*frdABCD*	Fermentation-deficient strain used as a base for complementation by rBOX expression	JBx_109929	2
CM15-TFA	CM15 **pSeva_TER_ACR** and **pBbA6a_fadAB**	Fermentation-deficient strain containing plasmid **pSeva_TER_ACR** and **pBbA6a_fadAB**	JBx_277738	This study
CM15-TA	CM15 **pSeva_TER_ACR**	Fermentation-deficient strain containing plasmid **pSeva_TER_ACR**	JBx_277739	This study
CM15-F	CM15 **pBbA6a_fadAB**	Fermentation-deficient strain containing plasmid **pBbA6a_fadAB**	JBx_277740	This study
CM15-TFA_{EnRx}	See Fig. 4 for genomic characterization.	Evolved CM15-TFA strain. Independent evolution replicate n from round x of ALE.		This study
CM15-TFA_{E2R18}	CM15-TFA *leuS* R502S *opgH* S523P *atoC* I129S TRC -10 T>C	Evolved CM15-TFA strain determined to be fastest growing and further characterized in bioreactor fermentations.	JBx_277741	This study
BL21 (DE3) FadA WT	BL21 (DE3) **pet28a_FadA_WT**	Expression strain containing plasmid **pet28a_FadA_WT**	JBx_277742	This study
BL21 (DE3) FadA Q59K	BL21 (DE3) **pet28a_FadA_Q59K**	Expression strain containing plasmid **pet28a_FadA_Q59K**	JBx_277743	This study
MG1655 *opgH* S523P	*E. coli* MG1655 *opgH* S523P	MG1655 strain with *opgH* S523P mutation introduced using MAGE	JBx_279789	This study
XL1-Blue	*E. coli* XL1-blue (*rec*A1 *end*A1 *gyr*A96 *thi*-1 *hsd*R17 *sup*E44 *rel*A1 lac [F´ *proAB lacI*^{q}*Z*Δ*M15* Tn*10* (Tet r)])	Used for DNA storage and preparation		Commercially available (Agilent)

Plasmid names are bolded

Supplementary Table 2: Plasmids used in this study

Plasmid name	Description	Registry #	Reference
**pSeva_TER_ACR**	Expression construct for *trans*-2-enoyl-CoA reductase (TER) from *Treponema denticola* and acyl-CoA reductase (ACR) from *Marinobacter aquaeolei* under control of the trc promoter	JBx_277729	This study
**pBbA6a_fadAB**	Expression construct for 3-ketoacyl-CoA thiolase (FadA) and Fatty acid oxidation complex subunit alpha (FadB) from *E. coli* under control of the LlacO-1 promoter.	JBx_277733	This study
**pET28a_FadA_WT**	Expression construct for 3-ketoacyl-CoA thiolase (FadA)	JBx_277735	This study
**pET28a_FadA_Q59K**	Expression construct for 3-ketoacyl-CoA thiolase (FadA) with Q59K substitution	JBx_277737	This study
**pORTMAGE-2**	Temperature-inducible Lambda Red/MutL expression plasmid for precise, high-efficiency MAGE. Ampicillin resistant.	Addgene 72677	[3]

Supplementary Table 3: Primers used in this study

Primer	Sequence
**BC1**	5’– cggtgtttgagcgggtttaaggatccaaactcgagtaagg –3’
**BC2**	5’– gtcgcctttgtaaagcatatgtatatctccttcttaaaagatcttttga –3’
**BC3**	5’– atccttactcgagtttggatccttaaacccgctcaaacacc –3’
**BC4**	5’– cttttaagaaggagatatacatatgctttacaaaggcgacac –3’
**BC5**	5’– atgcgtgggatttactggtaagactcctgttgatagatcca –3’
**BC6**	5’– ccattcgatggtgtcgacgtcggccgcgaattccagaaatc –3’
**BC7**	5’– agcgctttgaccgtatctaagcgtgtgtgaatcgcagaaa –3’
**BC8**	5’– ttaccagtaaatcccacgca –3’
**BC9**	5’– cagtagagagcgcgaggaacataaaccacagcggagcggggagataagacatcacgcccgtcaggaacaccgcacggtga –3’

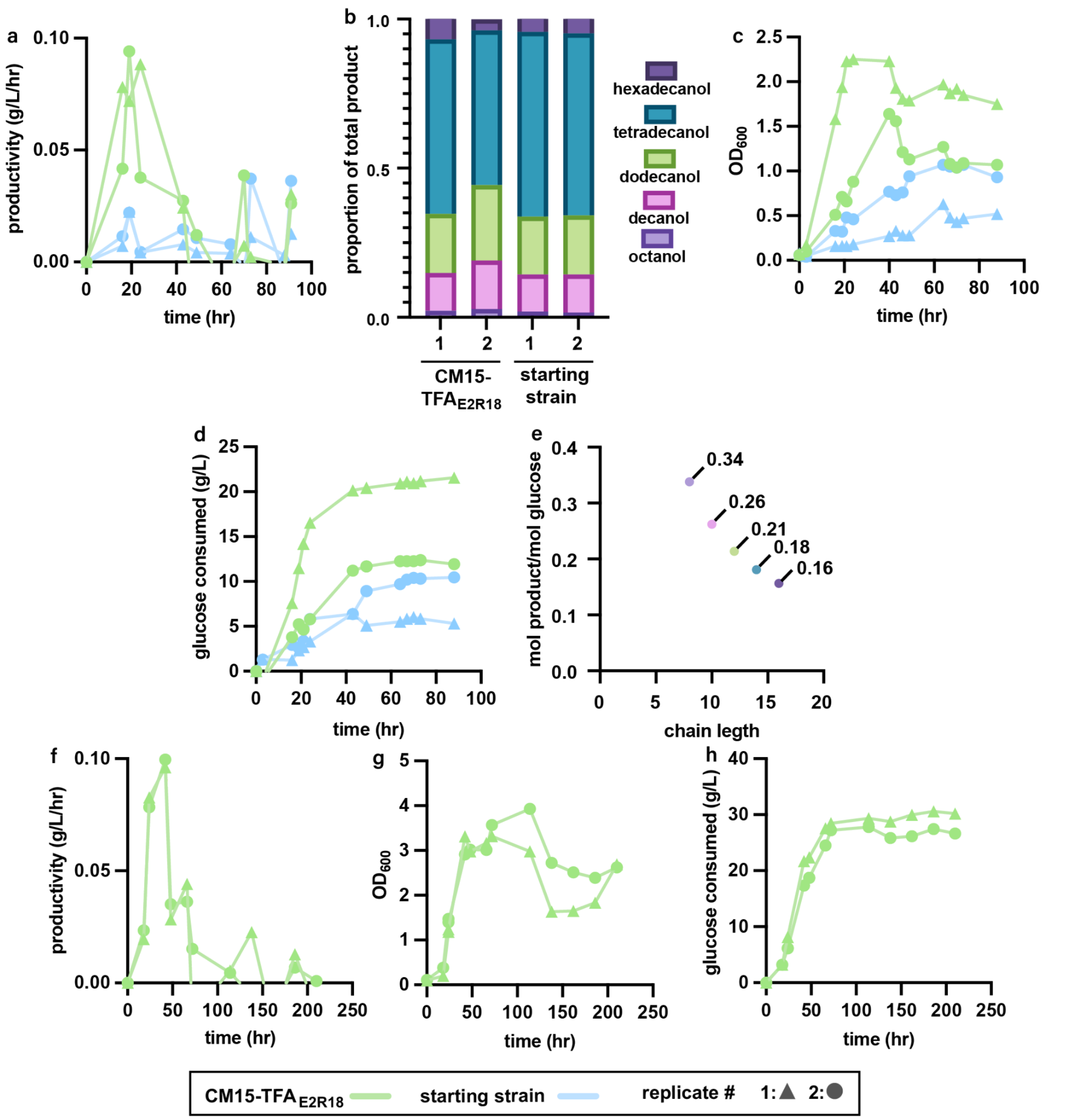

Supplementary Fig. 1 Bioreactor fatty-alcohol production by the starting strain CM15-TFA and evolved strain CM15-TFA$_{E2R18}$

a, Time course of fatty-alcohol productivity for bioreactors inoculated with either starting strain (CM15-TFA) or CM15-TFA$_{E2R18}$. **b,** For each bioreactor, fatty-alcohol composition is shown as the molar fraction of total fatty-alcohol titer contributed by each chain-length species. **c,** Optical density (OD_{600}) of each fermentation showing that CM15-TFA$_{E2R18}$ was able to reach a higher final OD_{600} than the starting strain (CM15-TFA). **d,** Glucose consumed over the course of fermentation in each bioreactor. **e,** Predicted molar production (yield coefficient) of assayed fatty alcohols per mol glucose. Theoretical yield predictions were generated by metabolic modeling using flux balance analysis. **f,** Time course of fatty-alcohol productivity for fermentations containing 20% dodecane overlay. **g,** Optical density (OD_{600}) of fermentations containing 20% dodecane overlay. **h,** Glucose consumed in fermentations containing 20% dodecane overlay. Bioreactor experiments used n = 2 biological replicate vessels per strain or condition; individual replicates are shown. Source data for this figure are provided in the Source Data file. Experiments were repeated 3 times.

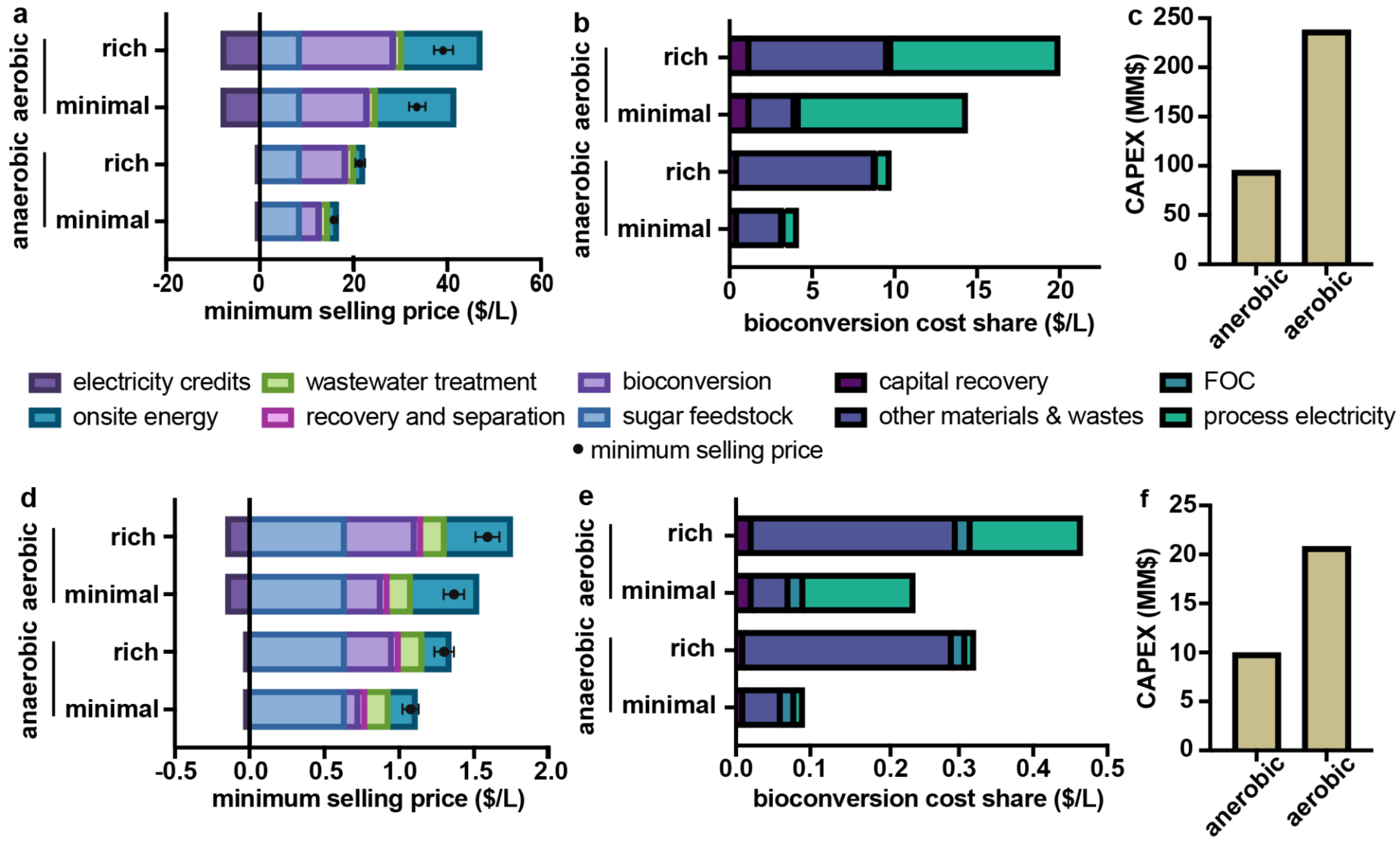

Supplementary Figure 2 Techno-economic analysis of fatty alcohol bioprocess
a, Minimum selling price of fatty alcohols at the current state of technology using either rich or minimal medium in an anaerobic or aerobic bioprocess. **b,** Expanded view of cost share of bioconversion of feedstocks to fatty alcohols at the current state of technology demonstrating the relative cost-effectiveness of an anaerobic process in minimal medium. **c,** Biorefinery total capital investment (CapEx) based on the current state of technology. **d,** Minimum selling price of fatty alcohols in an optimal future scenario using either rich or minimal medium in an anaerobic or aerobic bioprocess. **e,** Expanded view of cost share of bioconversion of feedstocks to fatty alcohols in an optimal future scenario demonstrating the relative cost-effectiveness of an anaerobic process in minimal medium. **f,** Biorefinery CapEx based on an optimal future scenario. FOC = Fixed operating cost (includes labor, maintenance, and property taxes). Source data for this figure are provided in the Source Data file.

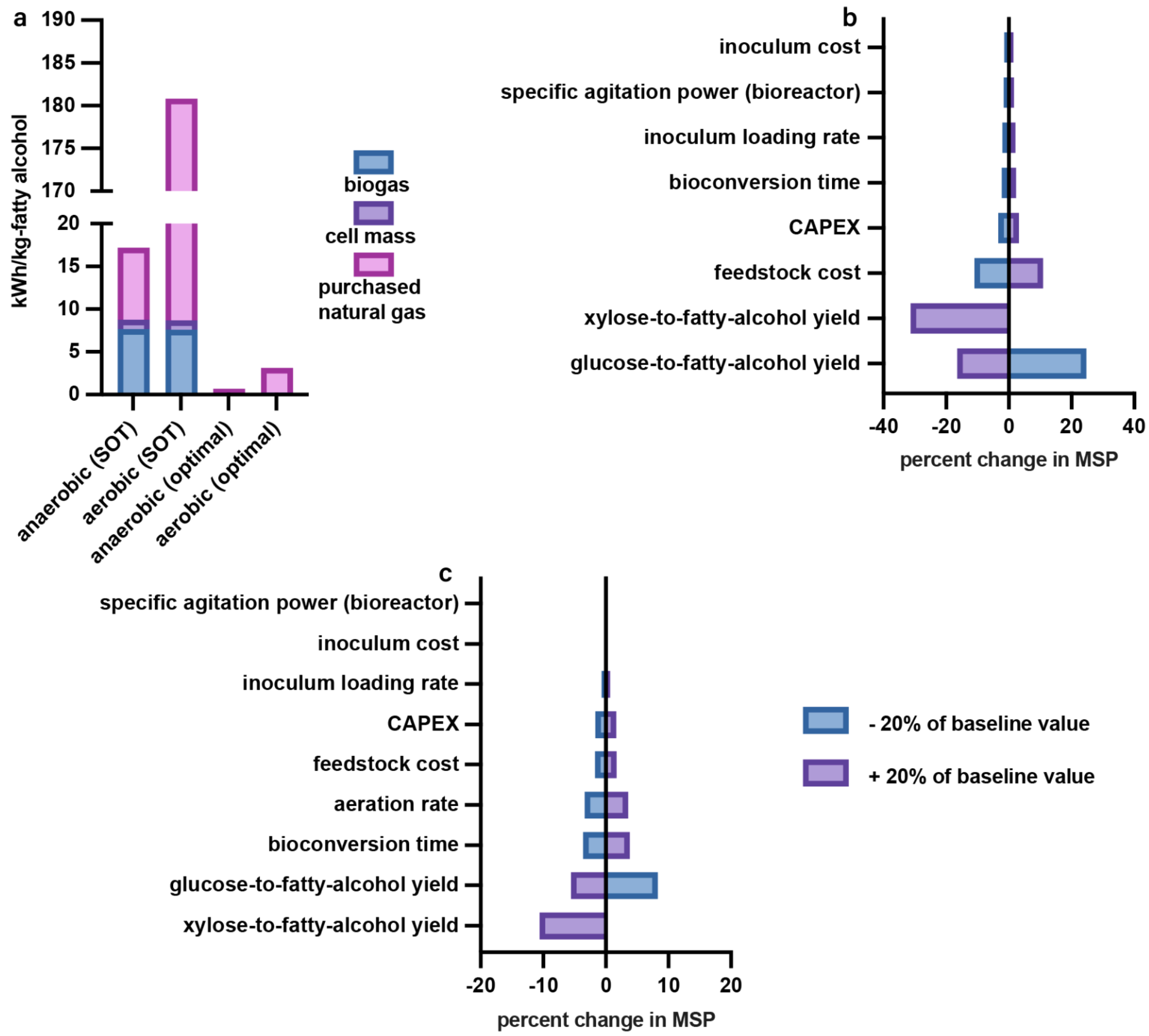

Supplementary Figure 3 Energy sources and sensitivity analysis results.
a, Contributions to biorefinery energy demand under both anaerobic and aerobic scenarios. **b**, Most influential parameters affecting the minimum selling price (MSP) for the anaerobic bioconversion process. **c**, Most influential parameters affecting the MSP for the aerobic bioconversion process. For the sensitivity analysis, parameters were varied by ±20% relative to their baseline values (Supplementary Data 1). Additionally, the xylose-to–fatty alcohol yield is assumed to be 90% of the glucose-based yield, with a minimum value of zero, reflecting that current experimental yields are based solely on a glucose feedstock. CAPEX = Total capital investment. Source data for this figure are provided in the Source Data file.

	-35 BOX / -10 BOX / lacO
**wildtype**	gctgttgacaattaatcatccggctcgtataatgtgtggaattgtgagcggataacaatttca
**1R6**	gctgttggcaattaatcatccggctcgtataatgtgtggaattgtgagcggataacaatttca
**1R12**	gctgttgacaattaatcatccggctcgtataacgtgtggaattgtgagcggataacaatttca
**1R18**	gctgttgacaattaatcatccggctcgtataacgtgtggaattgtgagcggataacaatttca
**2R6**	gctgttgataattaatcatccggctcgtataatgtgtggaattgtgagcggataacaatttca
**2R12**	gctgttgacaattaatcatccggctcgtataacgtgtggaattgtgagcggataacaatttca
**2R18**	gctgttgacaattaatcatccggctcgtataacgtgtggaattgtgagcggataacaatttca
**3R6**	gctgttggcaattaatcatccggctcgtataatgtgtggaattgtgagcggataacaatttca
**3R12**	gctgttggcaattaatcatccggctcgtataatgtgtggaattgtgagcggataacaatttca
**3R18**	gctgttggcaattaatcatccggctcgtataatgtgtggaattgtgagcggataacaatttca

Supplementary Figure 4 Mutations to TRC promoter of pSEVA_TER_ACR

Mutations to the TRC promoter of pSeva_TER_ACR are highlighted in red for each sequenced isolate from the ALE experiment. All mutations are located in either the -35 or -10 BOX of the TRC promoter. Sequences from n = 9 evolved isolates from three independent ALE lineages are shown. Source data for this figure are provided in the Genome Sequence Archive.

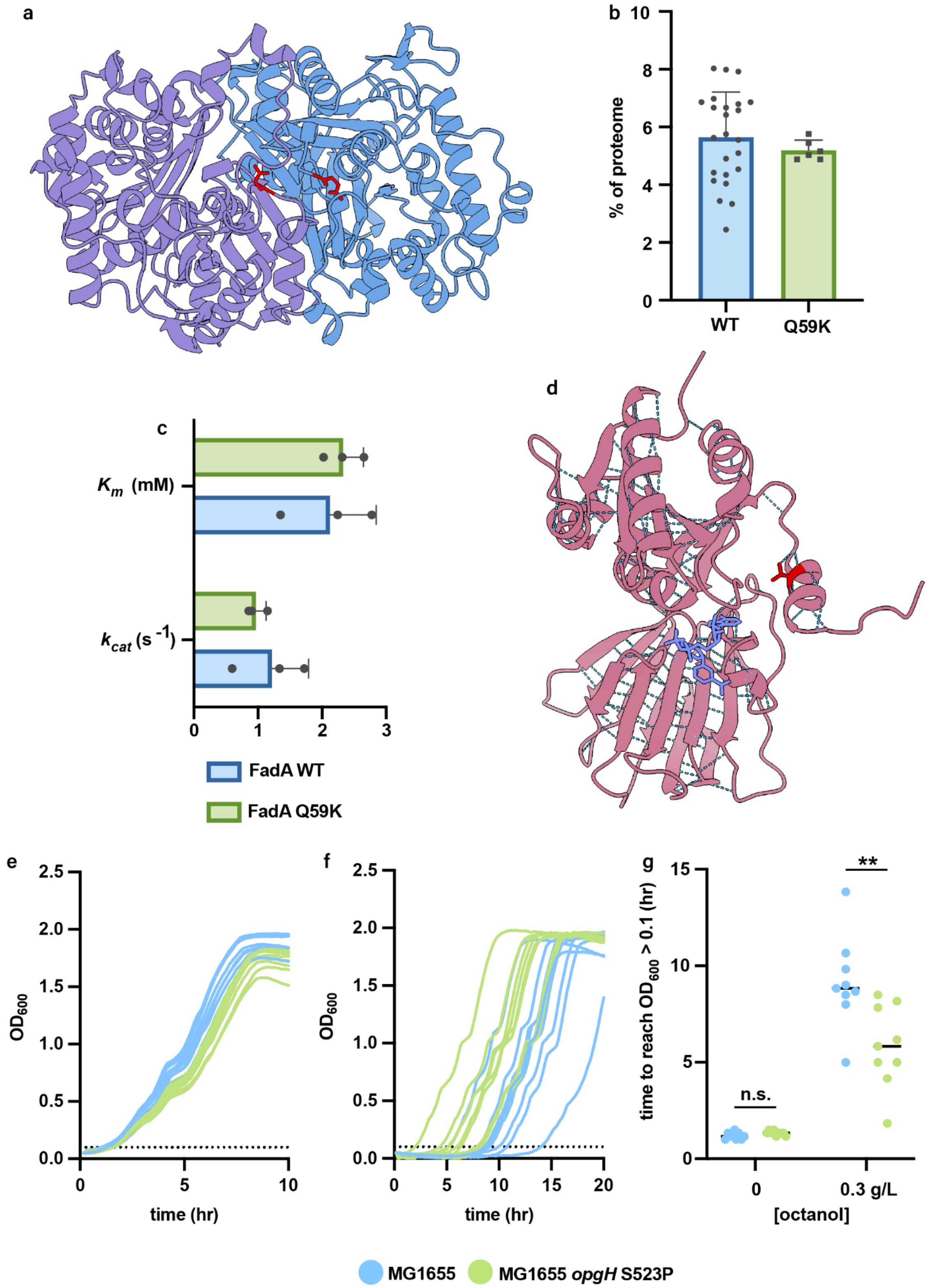

Supplementary Figure 5 Investigation of *fadA*, *nadK* and *opgH* mutations

a, Structural prediction of FadA homodimer (purple and blue chains) generated by Boltz-2x showing the location of Q59 in red (which was mutated to lysine in the later rounds of ALE lineage 3) at the dimer

interface. **b,** Protein abundance of FadA in ALE isolates containing the WT FadA enzyme (n=24) or the mutant FadA Q59K (n=6). **c,** K_m and k_{cat} determination of FadA WT and FadA Q59K for the thiolysis reaction of acetoacetyl-CoA (n=3). **d,** Structural prediction of NadK monomer (pale pink) with NADH bound (blue) generated by Boltz-2x showing the location of T280, which mutated to alanine in later rounds of ALE lineage 1. **e,** Growth of MG1655 and MG1655 *opgH* S523P in medium containing no octanol. (n=9) **f,** Growth of MG1655 and MG1655 opgH S523P in medium containing 0.3 g/L octanol. (n=9) **g,** Lag phase duration of cultures of MG1655 and MG1655 *opgH* S523P grown in medium containing 0.3 g/L octanol or no octanol. Lag phase duration is estimated by the point at which OD_{600} > 0.1. (n=9) Error bars represent s.d. Statistical comparisons in g were performed using unpaired, two-tailed Student's t-tests. Source data for this figure are provided in the Source Data file.

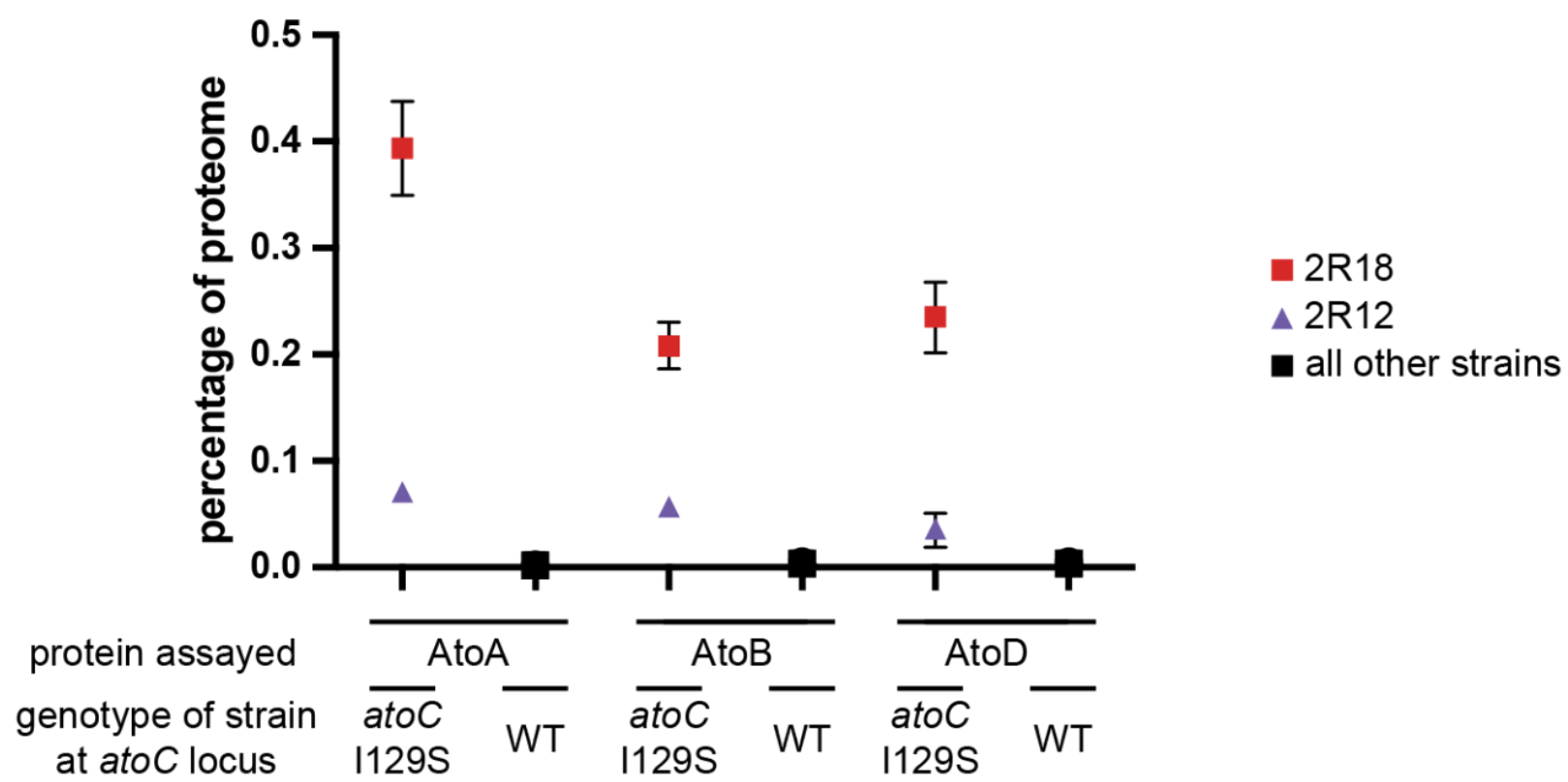

Supplementary Figure 6 Expression of proteins AtoA, AtoB and AtoD in strains containing *atoC* I129S

Expression of *ato* operon mediating short-chain fatty acid activation and β-oxidation in sequenced isolates of the ALE experiment. Expression of ato genes *atoA*, *atoB* and *atoD* is elevated in strains containing an I129S mutation to *atoC*, a characterized activator of the *ato* operon (n=3). Datapoints represent the mean and error bars represent the S.D. from the mean. Error bars represent s.d. Source data for this figure are provided in the Source Data file.

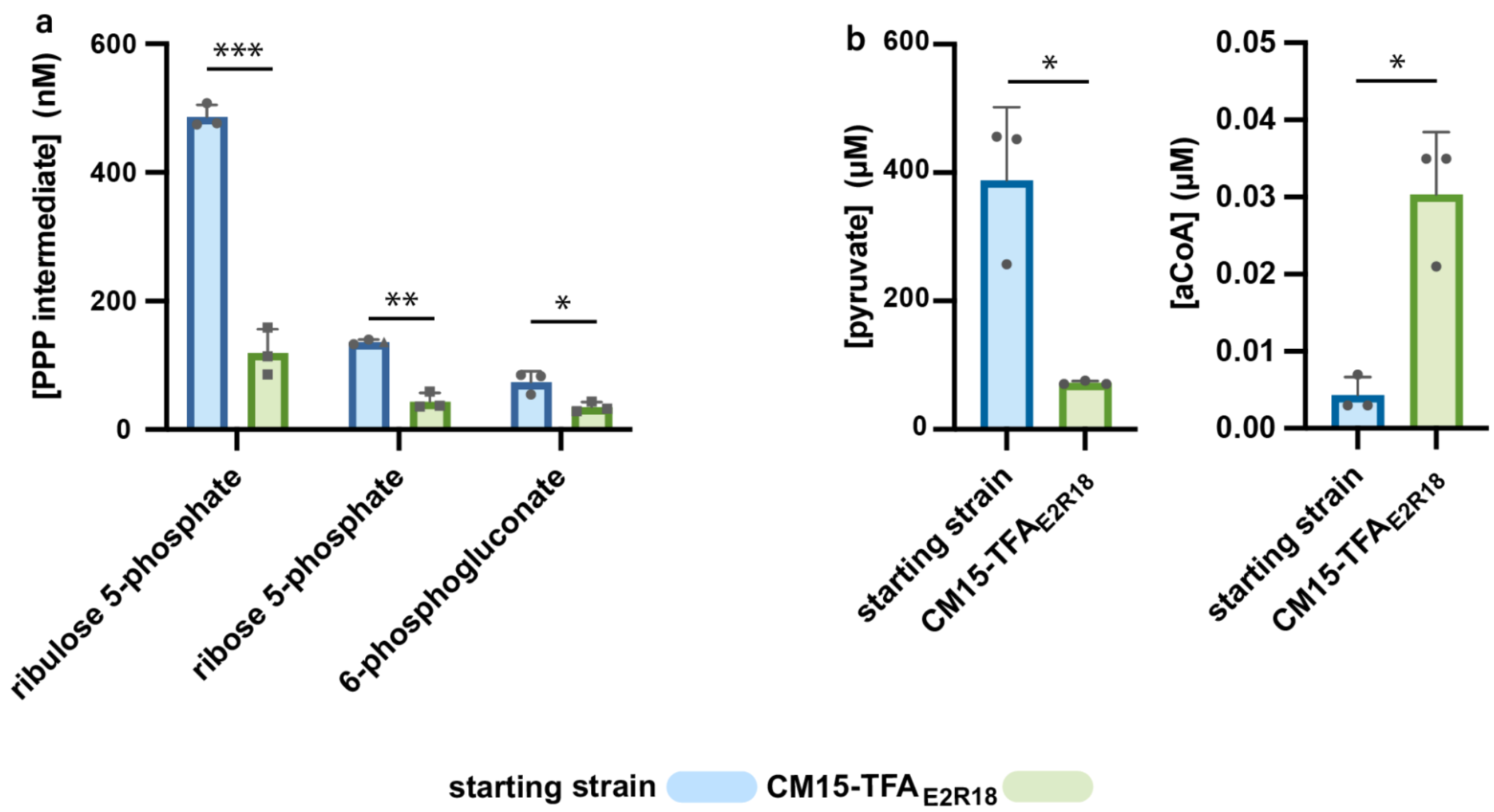

Supplementary Figure 7 Metabolomic analysis of starting and evolved CM15-TFA$_{E2R18}$ strain
a, Quantification of pentose phosphate pathway (PPP) intermediates in cell lysates showing decreased levels of ribulose 5-phosphate (p=0.0006), ribose 5-phosphate (p=0.004) and 6-phosphogluconate (p=0.03) in CM15-TFA$_{E2R18}$ (n=3). **b**, Quantification of substrate and product of pyruvate formate lyase (PFL) in cell lysates showing a decreased concentration of pyruvate (p=0.04) and increased concentration of acetyl-CoA (p=0.02) in CM15-TFA$_{E2R18}$ (n=3). Error bars represent s.d. Statistical comparisons were performed using unpaired, two-tailed Student's t-tests. Source data for this figure are provided in the Source Data file.

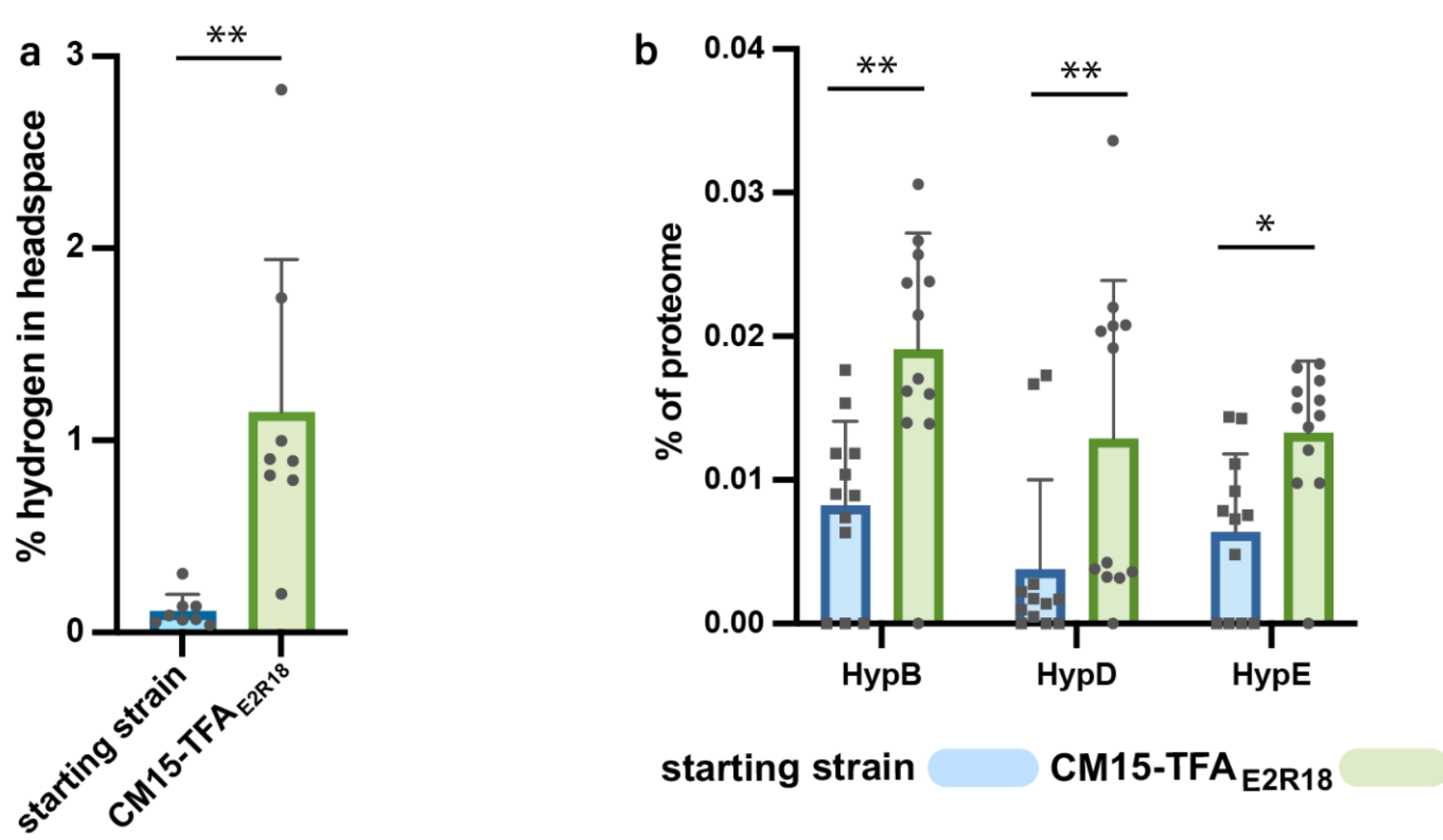

Supplementary Figure 8 Hydrogen production and hydrogenase subunit expression in the starting strain CM15-TFA and evolved strain CM15-TFA$_{E2R18}$
a, Hydrogen concentration in the headspace of sealed anaerobic vials containing cultures of CM15-TFA and evolved strain CM15-TFA$_{E2R18}$ after a 3-day fermentation (n=8). **b,** Protein abundance as the percentage of the total proteome occupied by hydrogenase maturation factors HypB/D/E (n=4 biological replicates assayed in triplicate). Error bars represent s.d. Statistical comparisons were performed using unpaired, two-tailed Student's t-tests. Source data for this figure are provided in the Source Data file.

Supplementary References

1. Blattner, F. R. *et al.* The complete genome sequence of Escherichia coli K-12. *Science* **277**, 1453–1462 (1997).
2. Mehrer, C. R., Incha, M. R., Politz, M. C. & Pfleger, B. F. Anaerobic production of medium-chain fatty alcohols via a β-reduction pathway. *Metab. Eng.* **48**, 63–71 (2018).
3. Nyerges, Á. *et al.* A highly precise and portable genome engineering method allows comparison of mutational effects across bacterial species. *Proc. Natl. Acad. Sci. U. S. A.* **113**, 2502–2507 (2016).
4. Baral, N. R. *et al.* Production cost and carbon footprint of biomass-derived dimethylcyclooctane as a high-performance jet fuel blendstock. *ACS Sustain. Chem. Eng.* **9**, 11872–11882 (2021).
5. Humbird, D. *et al.* Process design and economics for biochemical conversion of lignocellulosic biomass to ethanol. https://docs.nrel.gov/docs/fy11osti/47764.pdf.
6. Baral, N. *et al.* Techno-economic analysis and life-cycle greenhouse gas mitigation cost of five routes to bio-jet fuel blendstocks. *Energy Environ. Sci.* **12**, 807–824 (2019).
7. Davis, R. *et al.* Biochemical deconstruction and conversion of biomass to fuels and products via integrated biorefinery pathways. https://docs.nrel.gov/docs/fy19osti/71949.pdf.
8. Wholesale Price 99% N-dodecane Cas 112-40-3. *www.alibaba.com* https://www.alibaba.com/product-detail/Wholesale-Price-99-N-Dodecane-CAS_1601557605530.html?spm=a2700.7724857.0.0.3ae925d1yr78SR.
