# Supplementary material for: Replacing anaerobic fermentation with reverse β-oxidation enables growth-coupled evolution for enhanced fatty-alcohol production
Source: Nat Commun. 2026 Oct 14;17:11615. doi: 10.1038/s41467-026-78481-1 (PMC13754892; doi:10.1038/s41467-026-78481-1)
Supplement: Supplementary file 2 [file 41467_2026_78481_MOESM2_ESM.pdf]

Description of Additional Supplementary files

Title: Supplementary Data 1

Description:

The current state of technology (SOT) considered a delivered cost of lignocellulosic sugar of $615.5/bdt4 . The SOT scenario employed a 20% (v/v) dodecane overlay, which was recovered and reused, assuming an overall recovery of 95%. Dodecane was considered to have nearly 99% purity, with a bulk price of $2/kg.8 ϕThe SOT scenario cost is calculated assuming an inoculum loading rate of 10%. The SOT scenario cost is calculated based on the average titer of 3.75 g/L (114 hr timepoint) demonstrated in this study. The SOT scenario cost is calculated based on the average yield of 9.6 g per 100 g of glucose demonstrated in this study. The SOT scenario cost is calculated based on bioconversion time of 114 h demonstrated in this study. SOT scenario only utilizes glucose. The SOT scenario cost is calculated considering product recovery of 95%.
