# Supplementary material for: Replacing anaerobic fermentation with reverse β-oxidation enables growth-coupled evolution for enhanced fatty-alcohol production
Source: Nat Commun. 2026 Oct 14;17:11615. doi: 10.1038/s41467-026-78481-1 (PMC13754892; doi:10.1038/s41467-026-78481-1)
Supplement: Supplementary file 3 [file 41467_2026_78481_MOESM3_ESM.pdf]

## Supplementary Data 1: Major data inputs used to develop the techno-economic analysis model

| Parameter | Unit | Value | Notes |
|---|---|---|---|
| Biorefinery size | bone-dry metric ton (bdt) of sugars/day | 1000[4] | |
| Sugar feedstock cost | $/bdt | 220.9[4,α] | |
| **Sugar feedstock composition** | | | |
| **Glucose** | wt% | 58[4] | |
| **Xylose** | wt% | 42[4] | |
| **Bioconversion**[θ] | | | |
| **Anaerobic Process: CSTR reactor** | | | |
| **Bioreactor size** | $m^3$ (each) | 3785.41[5] | |
| **Reactor power** | $kW/m^3$ | 0.36[5] | |
| **Inoculum cost (minimal medium/rich medium)** | $/kg | 0.06/0.36 | This study |
| **Inoculum loading rate** | wt% | 5[φ5,6] | |
| **Corn steep liquor (CSL) loading** | wt% | 0.25[5,6] | |
| **Diammonium phosphate (DAP) loading** | g/L | 0.33[5,6] | |
| **CSL cost** | $/kg | 0.07[5,6] | |
| **DAP cost** | $/kg | 0.36[5,6] | |
| **Fatty alcohol titer** | g/L | 59.4[β] | Calculated based on 90% of theoretical yield |
| **Fatty alcohol yield** | % of stoichiometric theoretical yield | 90[γ] | Assumed |
| **Bioconversion time** | h | 24[δ] | Assumed based on the maximum productivity demonstrated in this study |
| **Glucose utilization to product** | - | Yes[λ] | This study |
| **Xylose utilization to product** | - | Yes[λ] | Assumed |
| **Aerobic Process: bubble column reactor** | | | |
| **Bioreactor size** | $m^3$ (each) | 1000[7] | |
| **Aeration rate** | vvm | 0.6[7] | |
| **Air pressure** | bar | 3.1[7] | |
| **Inoculum cost (minimal medium/rich medium)** | $/kg | 0.06/0.36 | Calculated in this study |
| **Inoculum loading rate** | wt% | 5[φ5,6] | |
| **Corn steep liquor (CSL) loading** | wt% | 0.25[5,6] | |
| **Diammonium phosphate (DAP) loading** | g/L | 0.33[5,6] | |
| **CSL cost** | $/kg | 0.07[5,6] | |
| **DAP cost** | $/kg | 0.36[5,6] | |
| **Fatty alcohol titer** | g/L | 59.4[β] | Assumed for comparison with anaerobic process |
| **Fatty alcohol yield** | % of stoichiometric theoretical yield | 90[γ] | Assumed for comparison with |

| | | | |
|---|---|---|---|
| | | | anaerobic process |
| **Bioconversion time** | h | 24[δ] | Assumed for comparison with anaerobic process |
| **Glucose utilization to product** | - | Yes[λ] | Assumed for comparison with anaerobic process |
| **Xylose utilization to product** | - | Yes[λ] | Assumed for comparison with anaerobic process |
| **Recovery and separation** | | | |
| **Fatty alcohol recovery** | wt% | 98[ϱ,5,6] | |
| **Wastewater treatment** | | | |
| **Nutrient for anaerobic digester** | $/kg | 0.45[5,6] | |
| **Nutrient loading rate** | wt% | 0.02[5,6] | |
| **Organic matter to biogas** | wt% | 86[5,6] | |
| **Onsite energy & utilities** | | | |
| **Boiler chemicals cost** | $/kg | 5[5] | |
| **Natural gas cost** | $/kg | 0.22[6] | |
| **Water** | $/kg | 0.00022[5] | |
| **Clean-in-place (CIP) cost** | $/kg | 0.35[5] | |
| **Electricity cost/credits** | $/kWh | 0.07[5] | |
