# Supplementary material for: Replacing anaerobic fermentation with reverse β-oxidation enables growth-coupled evolution for enhanced fatty-alcohol production
Source: Nat Commun. 2026 Oct 14;17:11615. doi: 10.1038/s41467-026-78481-1 (PMC13754892; doi:10.1038/s41467-026-78481-1)
Supplement: Supplementary file 5 [file 41467_2026_78481_MOESM5_ESM.pdf]

nature portfolio

Peer Review File

Replacing anaerobic fermentation with reverse β-oxidation enables growth-coupled evolution for enhanced fatty-alcohol production

Corresponding Author: Professor Jay Keasling

This file contains all reviewer reports in order by version, followed by all author rebuttals in order by version.

Version 1:

Reviewer comments:

Reviewer #1

(Remarks to the Author)

This study presents an innovative and highly practical metabolic engineering strategy. By replacing the native anaerobic fermentation pathway in E. coli with a reverse β-oxidation (rBOX) pathway, a growth-coupled fatty alcohol production system was successfully constructed. Furthermore, the authors exploited this growth-coupled strategy by employing adaptive laboratory evolution (ALE) to improve the growth and productivity of an rBOX strain in an untargeted manner. Ultimately, a fatty alcohol production of up to 4.0 g/L and a productivity of 110 mg/L/hr were achieved under anaerobic conditions. By combining proteomic and genomic analyses, the authors provided an in-depth elucidation of the altered enzyme expressions at key metabolic nodes and the activation mechanisms of potential genes within the evolved strains. This work not only demonstrates the potential of rBOX as a practical synthetic fermentation pathway but also provides an excellent paradigm for the development of non-native product chassis cells using growth-coupling strategies. Overall, this work is logically rigorous and highly innovative; however, it is recommended that the authors address the following points to further enhance the rigor of the manuscript.Comments:

Line 161: FadA is not labeled in Figure 1. Please add the annotation.

Line 258: The authors hypothesized that in this synthetic fermentation system, biomass accumulation is stoichiometrically linked to product formation. To support this hypothesis, the authors should provide the quantitative relationship between product titer and final OD in fed-batch fermentations (e.g., g product / g DCW or g product / OD) and compare the values before and after evolution.

Line 266: During the fed-batch fermentation process, the OD reached its maximum value of approximately 2.2 at 20 hours, with glucose consumption exceeding 15 g/L during this period (Supplementary Fig. 2c & 2d). This indicates that, compared to the small-scale fermentation system, the availability of the substrate glucose was effectively enhanced, thereby improving cell growth and production performance. Why not consider appropriately increasing the glucose feeding concentration or the feeding frequency?

Line 320: In Figure 3, productivity data is presented with n=2. Please clarify in the figure legend or Methods section the rationale for using two biological replicates.

Line 351: The authors sequenced and identified 10 unique genomic mutations, but functional validation of these mutations was not further conducted. For example, mutations in mrdA and ompH can regulate cell envelope biosynthesis and function. Is this directly related to fatty alcohol tolerance? Would overexpression of these mutants directly alleviate the product toxicity issue in the cells?

Line 512: The authors successfully constructed an anaerobic growth phenotype dependent on fatty alcohol production through a growth-coupled strategy. Is it possible to apply this 'redox-balanced growth-coupling' strategy to the biosynthesis of other high-value compounds?

(Remarks on code availability)

Reviewer #2

(Remarks to the Author)
I co-reviewed this manuscript with one of the reviewers who provided the listed reports. This is part of the Nature Communications initiative to facilitate training in peer review and to provide appropriate recognition for Early Career Researchers who co-review manuscripts.

(Remarks on code availability)

Reviewer #3

(Remarks to the Author)
This manuscript by Cawthon and Cowan et al., presents the development of an E. coli platform for fatty alcohol production under anaerobic conditions using a redox-balanced reverse β-oxidation (rBOX) pathway, together with strain improvement through growth-coupled adaptive laboratory evolution. Overall, I found the study interesting, well designed, and carefully executed. The manuscript is logically structured, and the authors provide detailed experimental validation across most stages of the work. In particular, the ALE and proteomic analyses offer a convincing and nuanced view of how rBOX function and redox balance were improved in the evolved strains. In addition, the inclusion of a techno-economic analysis enhances the broader significance of the study by helping to place the results in a practical biomanufacturing context. Taken together, the manuscript is suitable for publication in Nature Communications after minor revision.

1. It would be helpful for the readers if the authors briefly explain how the abbreviation of the TFAE2R18 strain name was derived. This would improve clarity, especially for those not immediately familiar with the naming logic used in the study.

2. Since the current system is based on plasmid expression, I would appreciate some discussion on the expected performance of a plasmid-free strain generated through genomic integration of the pathway genes. Even in the absence of experimental data, a brief discussion of the extent to which productivity or robustness might be maintained in such a system would add useful perspective, especially in relation to industrial implementation

3. The manuscript identifies FadB as an important rate-limiting step in the rBOX pathway. At the same time, depending on pathway design and flux balance, other steps such as those catalyzed by thiolase and TER (or acyl-CoA dehydrogenase) could also become limiting. For this reason, fine control of pathway strength and metabolic flux across the full rBOX module seems likely to be critical for maximizing fatty alcohol production.

In this context, the authors note that downregulation of TER appeared to be beneficial for growth. As the authors suggest, this may indicate that the upstream steps were not sufficiently optimized to support higher overall pathway flux. It would therefore be helpful if the authors could comment on whether further improvements in fatty alcohol production might be achieved by replacing upstream enzymes, including FadB, with homologs from other organisms, or by engineering these enzymes directly. If the authors have a view on which of these strategies may be more promising, a brief discussion would strengthen the manuscript.

4. In Figure 1, the green and blue boxes are useful for showing pathway organization. However, since several enzymes are also discussed in the main text, I would recommend labeling the enzyme names more explicitly in the figure. This would make the figure easier to follow and improve consistency between the figure and the manuscript text.

(Remarks on code availability)

Reviewer #4

(Remarks to the Author)
I have reviewed the techno-economic analysis section of the manuscript. Overall, the TEA is well integrated with the biological results and clearly highlights the potential economic advantages of anaerobic processing. The use of SuperPro Designer, minimum selling price (MSP) calculations, and comparative scenario analysis is appropriate and consistent with standard TEA practice. However, in its current form, the TEA lacks sufficient robustness and transparency to support publication in a high-impact journal. Key assumptions are insufficiently justified, and no uncertainty or sensitivity analysis is provided.
The following points should be addressed to strengthen the TEA:

1. The manuscript does not adequately disclose the underlying economic assumptions. These should be clearly presented, preferably in a summary table, to enable reproducibility and critical evaluation.
2. While the manuscript reports a substantial MSP reduction (~53%) for the anaerobic process, the robustness of this conclusion is not tested. No sensitivity or uncertainty analysis is presented. At a minimum, sensitivity to key parameters, such as fermentation titer, yield, feedstock cost, capital expenditure (CAPEX), and retention time, should be evaluated using ±20% variation. A tornado plot or spider plot is strongly recommended. This is critical, as the conclusion that “anaerobic processing is economically superior” may change under realistic uncertainty.
3. The future scenario assumes very aggressive performance targets, including 90% of theoretical conversion efficiency and high xylose utilization (90% of glucose efficiency). These assumptions are not supported by justification or benchmarking. The authors should either moderate these values or justify them using literature benchmarks (e.g., commercial ethanol plants or analogous fermentation pathways).
4. The TEA mentions modeling of a product mixture, but provides no meaningful discussion of downstream separation and purification steps. Key information is missing regarding separation strategy, recovery efficiencies, and associated costs. DSP often dominates overall process economics and should be described explicitly.
5. Although biogas recovery from wastewater treatment is included for energy recovery, no quantitative energy balance is presented. The authors should quantify the fraction of total plant energy demand offset by biogas and discuss the implications for operating costs and overall process sustainability.

(Remarks on code availability)

Version 2:

Reviewer comments:

Reviewer #1

(Remarks to the Author)
The authors have addressed reviewers' comments.

(Remarks on code availability)

Reviewer #2

(Remarks to the Author)
I co-reviewed this manuscript with one of the reviewers who provided the listed reports. This is part of the Nature Communications initiative to facilitate training in peer review and to provide appropriate recognition for Early Career Researchers who co-review manuscripts.

(Remarks on code availability)

Reviewer #3

(Remarks to the Author)
I have carefully reviewed the revised manuscript focusing particularly on the enzymes involved in reverse β-oxidation. The authors have addressed all of my previous concerns in this area. I have no further comments and recommend acceptance of the manuscript for publication.

(Remarks on code availability)

Reviewer #4

(Remarks to the Author)
The authors have addressed all the comments raised. The reviewer has no further comments.

(Remarks on code availability)

Open Access This Peer Review File is licensed under a Creative Commons Attribution 4.0 International License, which permits use, sharing, adaptation, distribution and reproduction in any medium or format, as long as you give appropriate credit to the original author(s) and the source, provide a link to the Creative Commons license, and indicate if changes were made.
In cases where reviewers are anonymous, credit should be given to 'Anonymous Referee' and the source.
The images or other third party material in this Peer Review File are included in the article's Creative Commons license, unless indicated otherwise in a credit line to the material. If material is not included in the article's Creative Commons license and your intended use is not permitted by statutory regulation or exceeds the permitted use, you will need to obtain permission directly from the copyright holder.
To view a copy of this license, visit https://creativecommons.org/licenses/by/4.0/

REVIEWER COMMENTS

Reviewer #1 (Remarks to the Author):

This study presents an innovative and highly practical metabolic engineering strategy. By replacing the native anaerobic fermentation pathway in E. coli with a reverse β-oxidation (rBOX) pathway, a growth-coupled fatty alcohol production system was successfully constructed. Furthermore, the authors exploited this growth-coupled strategy by employing adaptive laboratory evolution (ALE) to improve the growth and productivity of an rBOX strain in an untargeted manner. Ultimately, a fatty alcohol production of up to 4.0 g/L and a productivity of 110 mg/L/hr were achieved under anaerobic conditions. By combining proteomic and genomic analyses, the authors provided an in-depth elucidation of the altered enzyme expressions at key metabolic nodes and the activation mechanisms of potential genes within the evolved strains. This work not only demonstrates the potential of rBOX as a practical synthetic fermentation pathway but also provides an excellent paradigm for the development of non-native product chassis cells using growth-coupling strategies. Overall, this work is logically rigorous and highly innovative; however, it is recommended that the authors address the following points to further enhance the rigor of the manuscript.

Thank you for carefully reviewing our manuscript. We appreciate your time and effort in carefully analyzing the work we present here. We believe that your comments and suggestions have helped us to further improve the manuscript and we are grateful for your feedback. Please see our responses to your comments below.

Comments:

Line 161: FadA is not labeled in Figure 1. Please add the annotation.

Thank you for identifying this omission. It has now been corrected.

Line 258: The authors hypothesized that in this synthetic fermentation system, biomass accumulation is stoichiometrically linked to product formation. To support this hypothesis, the authors should provide the quantitative relationship between product titer and final OD in fed-batch fermentations (e.g., g product / g DCW or g product / OD) and compare the values before and after evolution.

Thank you for identifying the lack of support for this hypothesis. In response to your suggestion we have quantified the relationship between product titer and final OD in fed-batch fermentation. The results (below) show that in fed-batch the evolved strain produced a higher amount of product per unit biomass, though this difference was not statistically significant at the timepoints assayed except for one. We believe that claiming the strains have a strict stoichiometric relationship between growth and titer universally was an overstatement and is not sufficiently supported by the data we have collected. We have chosen to edit the manuscript to remove the discussion of titer/ OD ratio and instead keep the manuscript focused around more traditional fermentation metrics (titer, rate and

yield). There seems to be a significant amount of variability in the titer per OD metric between different culture methods (tube vs bioreactor). Furthermore, the titer/ OD metric is confounded by cell death and lysis which is difficult to control for, and may explain why there is substantial variability between culture modes. For example there may be more cell death for the evolved strain in fed-batch conditions because it is generating a larger amount of toxic fatty alcohols (Supplementary Figure 1c shows decreases in OD for only the evolved strain around the 40 hour mark). Overall these reasons make us hesitant to present the titer/OD metrics in the revised manuscript. Accordingly, we have reorganized the manuscript to remove Fig. 2c and instead present the data from Supplementary Figure 1 (optical density and glucose consumed) in main text Figure 2. The text has been updated to reflect this change and remove discussion of titer produced per OD.

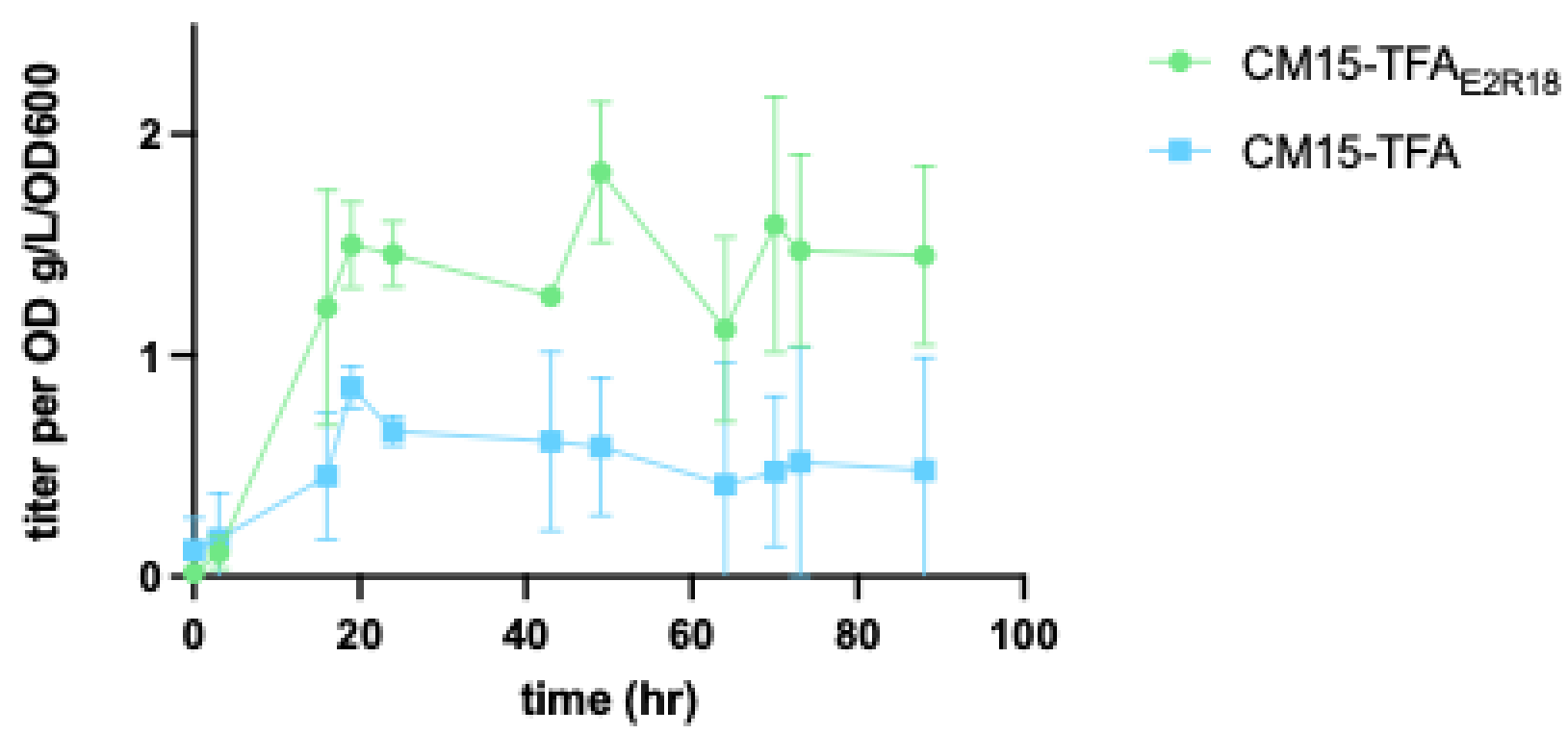

Line 266: During the fed-batch fermentation process, the OD reached its maximum value of approximately 2.2 at 20 hours, with glucose consumption exceeding 15 g/L during this period (Supplementary Fig. 2c & 2d). This indicates that, compared to the small-scale fermentation system, the availability of the substrate glucose was effectively enhanced, thereby improving cell growth and production performance. Why not consider appropriately increasing the glucose feeding concentration or the feeding frequency?

Thank you for this comment. In the bioreactor experiments the strains were able to consume much more glucose than in the small-scale fermentations. However, both experiments started at the same initial concentration of approximately 2% glucose. At the noted 20-hour timepoint there was still over 5 g/L of glucose remaining in the bioreactor vessel and additional glucose was fed shortly thereafter. 2% glucose is the standard concentration used by our group for cultivation of microorganisms in minimal medium. Glucose concentrations beyond 2% often result in overflow metabolism and can have deleterious effects on growth. In response to this comment we have assayed the growth of CM15-TFA$_{E2R18}$ in higher concentrations of glucose to ensure that our assumption that the standard 2% concentration of glucose was optimal for growth. The results

below show that higher concentrations of glucose do indeed inhibit the growth of CM15-TFA$_{E2R18}$ and also do not result in higher final ODs.

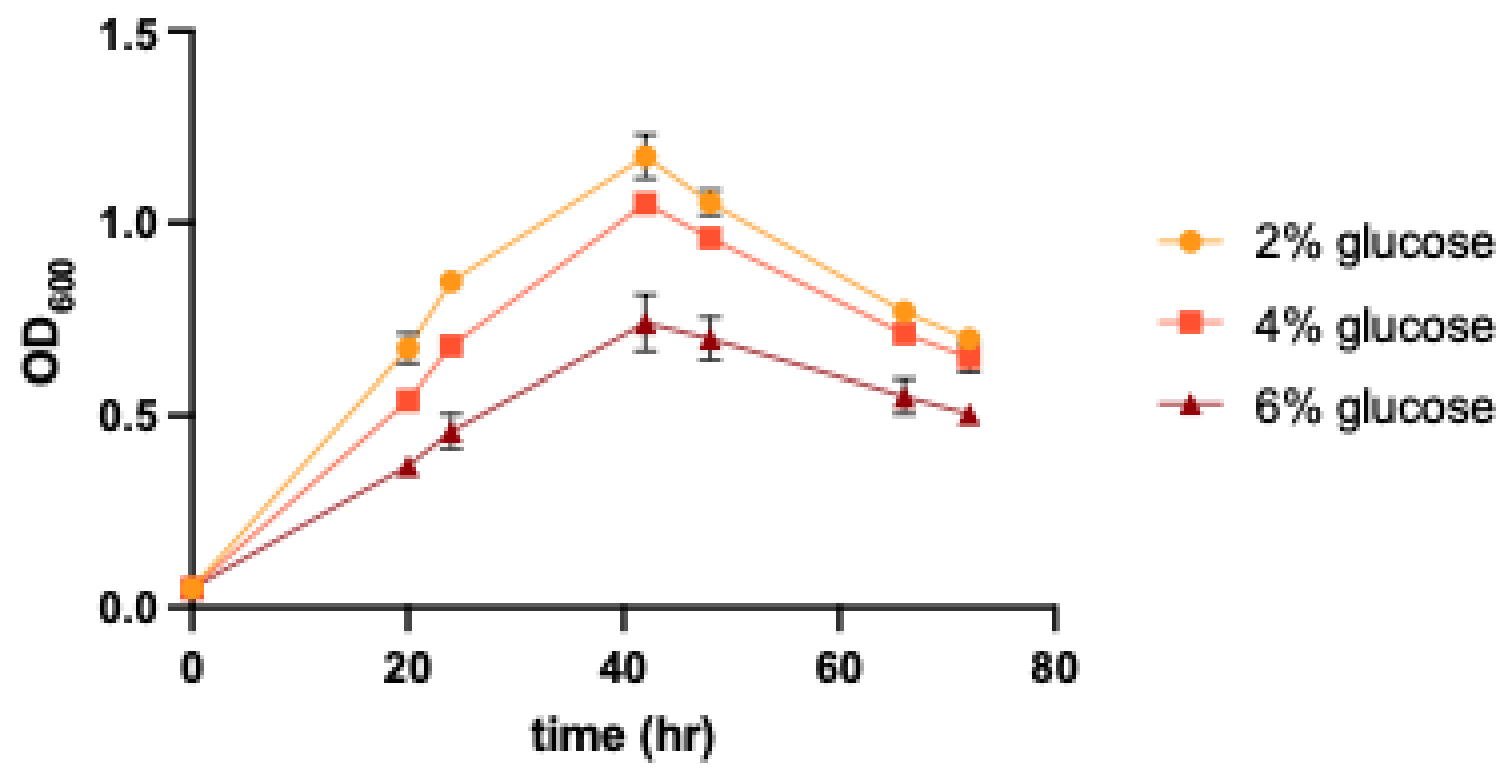

Line 320: In Figure 3, productivity data is presented with n=2. Please clarify in the figure legend or Methods section the rationale for using two biological replicates.

Thank you for your comment. Our Eppendorf DASGIP bioreactor system used for fed-batch fermentation has 4 total reactors that can be monitored in a single run. To minimize external factors which may have impacted the experiment-to-experiment variability (growth medium batch, duration of preculture, etc.), we chose to run the experiment in duplicate so that we could assay the CM15-TFA and CM15-TFA$_{E2R18}$ strains truly in parallel. To verify our results, we repeated this experiment more than 3 times and each time observed a similar magnitude of enhanced fatty alcohol production in CM15-TFA$_{E2R18}$ relative to CM15-TFA. We have updated the Methods section to explain this rationale.

“Fed-batch fermentations were performed in a four-vessel Eppendorf DASGIP bioreactor system. When two strains or conditions were compared in parallel within the same run, the system capacity limited the number of biological replicates to n = 2 per group. Thus, productivity measurements in Fig. 3 were obtained from duplicate bioreactor vessels for each strain/condition.”

Line 351: The authors sequenced and identified 10 unique genomic mutations, but functional validation of these mutations was not further conducted. For example, mutations in *mrdA* and *ompH* can regulate cell envelope biosynthesis and function. Is this directly related to fatty alcohol tolerance? Would overexpression of these mutants directly alleviate the product toxicity issue in the cells?

Thank you for this insightful comment. We first note that this comment helped us identify and correct a typo in the manuscript: the mutated gene is *opgH*, not *ompH*; both of these genes are involved in the stability and integrity of the bacterial membrane and were confused sometime during the writing process. The table in Fig. 4 showed the correct gene name *opgH*, however the text

incorrectly referred to this gene as *ompH*. We have corrected this error in the text and carefully reviewed all the other gene names to ensure no other errors are present.

We agree that the identified mutations in *opgH* and *mrdA* are biologically plausible candidates for improved fatty alcohol tolerance, as both genes are linked to cell envelope homeostasis. Because fatty alcohols are membrane-active molecules, mutations affecting envelope structure or stress response could reasonably contribute to improved tolerance in the evolved strain.

Following your suggestion we developed overexpression plasmids for both the mutant enzymes by cloning them from the evolved strains' genomes into expression vectors containing a P15a origin and lacUV5 promoter. However, these overexpression constructs gave inconsistent results and appeared to inhibit growth which we believe was due to the expression burden of these plasmids, which were likely greatly overexpressing OpgH and MrdA relative to their native expression levels.

In order to assay the observed mutations at the native expression level and transcriptional context we instead chose to use Multiplex Automated Genome Engineering (MAGE) to introduce only the single point mutations into the otherwise wild-type MG1655 base strain. We successfully generated a stable clonal population of MG1655 *opgH* S523P cells, however we were unable to stably generate the MrdA mutant in MG1655 despite repeated attempts to do so.

Using the MG1655 *opgH* S523P strain we assayed for improved tolerance to fatty alcohols. We found that the main chain lengths of fatty alcohols observed in our fermentation were not highly toxic to E. coli even at high concentrations in aqueous medium. We reasoned that this was due to their low solubility in water. Even though the absolute concentration in the medium may have been high, the majority would be in an insoluble fraction and would not impact the growth of the cells. Accordingly we used a shorter chain length alcohol, octanol, which is more soluble in water to model the effect of fatty alcohol toxicity instead of using the chain lengths produced in the manuscript.

We observed a mild, but reproducible shortening of the duration of the lag phase of the MG1655 *opgH* S523P strain when grown in 0.3 g/L octanol, which was not present in the absence of octanol. The results are summarized below and are shown in Supplementary Fig. 5.

This validation required substantial optimization, including generation of single-mutant MG1655 strains and selection of an informative fatty-alcohol chain length and concentration. We interpret the *opgH* S523P effect as reproducible but modest relative to other evolved mutations. We hope these additional experiments satisfactorily address the reviewer's concern.

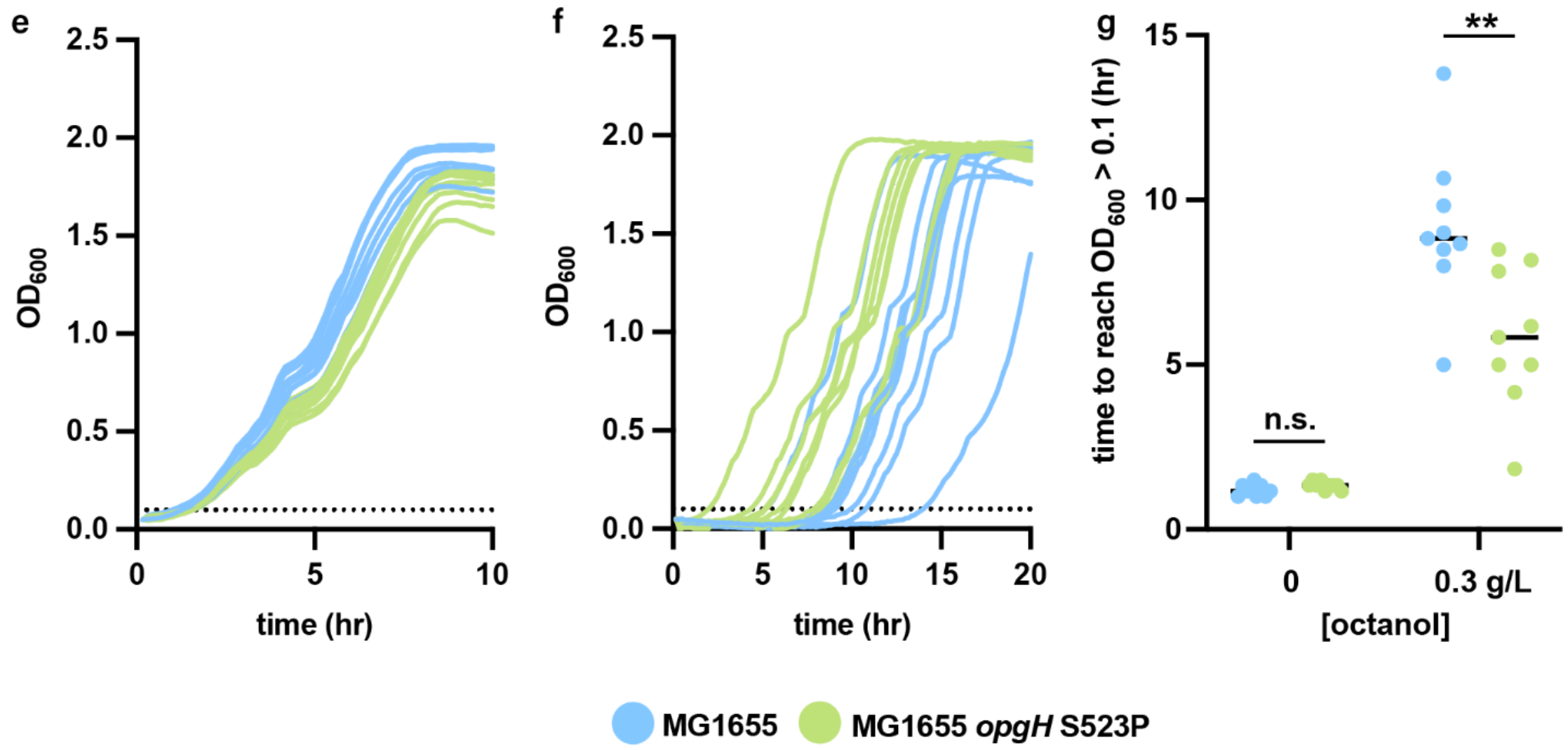

Supplementary Fig. 5 (relevant information only). e, Growth of MG1655 and MG1655 *opgH* S523P in medium containing no octanol. **f,** Growth of MG1655 and MG1655 opgH S523P in medium containing 0.3 g/L octanol. **g,** Lag phase duration of cultures of MG1655 and MG1655 *opgH* S523P grown in medium containing 0.3 g/L octanol or no octanol. Lag phase duration is estimated by the point at which OD_{600} > 0.1.

Line 512: The authors successfully constructed an anaerobic growth phenotype dependent on fatty alcohol production through a growth-coupled strategy. Is it possible to apply this 'redox-balanced growth-coupling' strategy to the biosynthesis of other high-value compounds?

Yes! Absolutely. This is the focus of ongoing work and advances in this area are described in an article we have published during this review process (10.1016/j.ymben.2026.05.004). The focus of this article is expanding redox-balance based growth coupling techniques to less reduced products than ethanol or fatty alcohols. We identify stoichiometric constraints to using redox-balance based growth coupling techniques for less reduced products and remedy these constraints by devising a carbon co-feeding strategy where glucose is used for growth and a secondary carbon source (acetate, propionate, etc.) is used only as a substrate for production (and not growth). Currently, selective pressures in this co-feeding strategy are less stable over long periods of time making ALE difficult or impossible. However, we are working to develop a more stable selective strain which can utilize and activate secondary carbon sources which would be more amenable to an ALE-type strategy which we have shown to be effective for rBOX. The following text has been added to the discussion to address your question.

“More broadly, growth coupling combined with adaptive evolution could provide a straightforward and low-cost route to improving strains that produce diverse bioproducts. However, redox-balance-based growth coupling carries inherent stoichiometric constraints, which can limit its suitability for products that do not consume sufficient reducing equivalents to serve as effective

redox sinks. Pathways whose redox and carbon stoichiometries differ from native fermentation pathways may therefore require additional design strategies."

Reviewer #2 (Remarks to the Author):

I co-reviewed this manuscript with one of the reviewers who provided the listed reports. This is part of the Nature Communications initiative to facilitate training in peer review and to provide appropriate recognition for Early Career Researchers who co-review manuscripts.

Reviewer #3 (Remarks to the Author):

This manuscript by Cawthon and Cowan et al., presents the development of an E. coli platform for fatty alcohol production under anaerobic conditions using a redox-balanced reverse β-oxidation (rBOX) pathway, together with strain improvement through growth-coupled adaptive laboratory evolution. Overall, I found the study interesting, well designed, and carefully executed. The manuscript is logically structured, and the authors provide detailed experimental validation across most stages of the work. In particular, the ALE and proteomic analyses offer a convincing and nuanced view of how rBOX function and redox balance were improved in the evolved strains. In addition, the inclusion of a techno-economic analysis enhances the broader significance of the study by helping to place the results in a practical biomanufacturing context. Taken together, the manuscript is suitable for publication in Nature Communications after minor revision.

Thank you for your thorough review of our manuscript. We sincerely appreciate the time and effort you devoted to evaluating our work. Your comments and suggestions have been valuable in helping us further strengthen the manuscript, and we are grateful for your feedback. Our point-by-point responses are provided below.

1. It would be helpful for the readers if the authors briefly explain how the abbreviation of the TFAE2R18 strain name was derived. This would improve clarity, especially for those not immediately familiar with the naming logic used in the study.

Thank you for this suggestion. We agree that our explanation of the naming convention was lacking. The structure of ExRy for naming evolved isolates is as follows: 'Ex' denotes the specific ALE replicate number (1, 2, or 3), 'Ry' denotes the round of evolution. We updated the manuscript to clarify the nomenclature.

"The strain which produced the highest titer and grew the best in small-scale fermentations was from passage round 18 of ALE replicate number 2 and was named CM15-TFA_{E2R18} (E2R18 denoting evolution replicate 2 and R18 denoting passage round 18)."

2. Since the current system is based on plasmid expression, I would appreciate some discussion on the expected performance of a plasmid-free strain generated through genomic integration of the pathway genes. Even in the absence of experimental data, a brief discussion of the extent to which

productivity or robustness might be maintained in such a system would add useful perspective, especially in relation to industrial implementation

Thank you for your comment. We agree that genomic integration of the pathway would likely improve the industrial relevance of this system by eliminating the need for antibiotics, improving genetic stability, and reducing the risk of plasmid loss during extended fermentation. These features would be expected to enhance robustness and reduce process cost. However, we also note that moving to chromosomal integration, particularly at a single-copy level, would probably decrease pathway expression compared to the current plasmid-based setup. This reduction could therefore lower productivity unless the integrated pathway undergoes further optimization.

We have added the following text to the discussion section to make these advantages and concerns clear:

"The technological readiness of the production chassis described here could be further improved through genomic integration of the pathway enzymes. Provided that single-copy genomic integration enables sufficient expression of the rBOX pathway genes, this approach could enhance strain and production stability, reduce cell-to-cell variability, and eliminate the need for antibiotics, thereby decreasing process costs and improving industrial relevance."

3. The manuscript identifies FadB as an important rate-limiting step in the rBOX pathway. At the same time, depending on pathway design and flux balance, other steps such as those catalyzed by thiolase and TER (or acyl-CoA dehydrogenase) could also become limiting. For this reason, fine control of pathway strength and metabolic flux across the full rBOX module seems likely to be critical for maximizing fatty alcohol production.

In this context, the authors note that downregulation of TER appeared to be beneficial for growth. As the authors suggest, this may indicate that the upstream steps were not sufficiently optimized to support higher overall pathway flux. It would therefore be helpful if the authors could comment on whether further improvements in fatty alcohol production might be achieved by replacing upstream enzymes, including FadB, with homologs from other organisms, or by engineering these enzymes directly. If the authors have a view on which of these strategies may be more promising, a brief discussion would strengthen the manuscript.

This is an interesting line of questioning. Our discussion of FadB expression was intended to highlight that the observed changes in gene expression, specifically the correlation between increased FadB expression and increased growth rate, were consistent with previous reports identifying FadB as the rate-limiting enzyme. (https://pubs.acs.org/doi/10.1021/acscatal.3c00379?ref=PDF) However, a more nuanced

interpretation is that calling an enzyme "rate limiting" simply reflects the fact that the initial expression level, or catalytic rate, was insufficient relative to the rest of the pathway. A more meaningful observation is that ALE both increased and decreased expression of pathway enzymes in order to balance expression and maximize flux, and that the increased FadB expression observed in our study is consistent with previous reports showing that higher FadB concentration led to an increased rate of product formation.

The specific homologs used in this study were chosen based on extensive prior work (Mehrer, 2018 https://doi.org/10.1016/j.ymben.2018.05.011), which identified these homologs as the most active for the production of long-chain fatty alcohols. There are also ongoing efforts to improve enzyme fitness through directed evolution. However, because many of these enzymes are carrying out their native reaction, although in reverse, it remains unclear whether variants can be generated that are more active than those produced through natural selection.

The text has been modified to better highlight the difficulty of achieving the optimal expression level for each rBOX enzyme and the importance of our selection scheme in naturally identifying an optimal balance of pathway enzyme expression.

“These results suggest that, while protein expression from the initial construct was sufficient to enable growth of CM15, the selection scheme fine-tuned gene expression across the rBOX pathway, downregulating genes expressed above their optimal level and upregulating genes that may previously have limited flux. These results highlight a key advantage of this growth-coupled evolution technique, which appears to be able to fine-tune enzyme expression across the entire rBOX pathway to maximize flux, reducing the need for targeted intervention to circumvent pathway bottlenecks.”

4. In Figure 1, the green and blue boxes are useful for showing pathway organization. However, since several enzymes are also discussed in the main text, I would recommend labeling the enzyme names more explicitly in the figure. This would make the figure easier to follow and improve consistency between the figure and the manuscript text.

This is a good suggestion to improve clarity. We have ensured that all the enzymes in the rBOX pathway (FadA, FadB, TER and ACR) are now labeled and all of the key fermentation gene knockouts (FRD complex, AdhE and LdhA) are labeled. Pyruvate formate lyase (PFL) is also mentioned later in the manuscript so we have added a label for PFL as well. We hope these adjustments address your concerns and improve the clarity of Fig. 1a.

Reviewer #4 (Remarks to the Author):

I have reviewed the techno-economic analysis section of the manuscript. Overall, the TEA is well integrated with the biological results and clearly highlights the potential economic advantages of anaerobic processing. The use of SuperPro Designer, minimum selling price (MSP) calculations, and comparative scenario analysis is appropriate and consistent with standard TEA practice.

However, in its current form, the TEA lacks sufficient robustness and transparency to support publication in a high-impact journal. Key assumptions are insufficiently justified, and no uncertainty or sensitivity analysis is provided.

Thank you for your thoughtful comments and valuable feedback. We believe that your feedback has allowed us to make a stronger manuscript and hope you agree that we have addressed all concerns raised. To make the full details of our model accessible to everyone, we have exported our model to the Standard Flowsheet Format (SFF, schema available here: https://sustainability-software-lab.github.io/pisces-standard-flowsheet-format/). The downloadable SFF version of our model is included in the Supporting Information and it is also posted to our recently launched web platform (https://projectpisces.org/flowsheets/103). Additionally, we have added sensitivity analyses of both aerobic and anaerobic processes with minimal medium as representative cases (Supplementary Figure 3b-c).

The following points should be addressed to strengthen the TEA:

1. The manuscript does not adequately disclose the underlying economic assumptions. These should be clearly presented, preferably in a summary table, to enable reproducibility and critical evaluation.

This is an important point. We have been working to improve transparency in the TEA and have now provided a downloadable JSON file containing all unit process and flow data in a standard flowsheet format as part of the SI, and also posted it to the Project PISCES database (https://projectpisces.org/flowsheets/103).

2. While the manuscript reports a substantial MSP reduction (~53%) for the anaerobic process, the robustness of this conclusion is not tested. No sensitivity or uncertainty analysis is presented. At a minimum, sensitivity to key parameters, such as fermentation titer, yield, feedstock cost, capital expenditure (CAPEX), and retention time, should be evaluated using ±20% variation. A tornado plot or spider plot is strongly recommended. This is critical, as the conclusion that “anaerobic processing is economically superior” may change under realistic uncertainty.

We appreciate the reviewer's suggestion. Accordingly, we performed sensitivity analyses of both aerobic and anaerobic processes with minimal media as representative examples. The results are presented in the revised Supplementary Figure 3b,c, which illustrates the percentage change in the minimum selling price of fatty alcohols when input parameters are varied by ±20%.

3. The future scenario assumes very aggressive performance targets, including 90% of theoretical conversion efficiency and high xylose utilization (90% of glucose efficiency). These assumptions are not supported by justification or benchmarking. The authors should either moderate these values or justify them using literature benchmarks (e.g., commercial ethanol plants or analogous fermentation pathways).

We agree that the assumed performance targets are aggressive. However, they are intended to represent an upper-bound grounded in literature benchmarks of commercial ethanol fermentation.

Given rBOX's bioenergetic equivalence to ethanol fermentation (same number of carbons and electrons needed to produce 1 ethanol or extend a fatty alcohol by two carbons) we believe this is an appropriately optimistic future scenario. In commercial corn ethanol processes, glucose-to-ethanol conversion efficiencies are nearly 90–95% theoretical[1,2], which are routinely achieved under optimized conditions. Similarly, lactic acid fermentation systems have demonstrated yields approaching theoretical limits (typically >85–90%) for hexose sugars[2]. We have revised the manuscript to better support the assumptions underlying our future case scenario and have pasted the text below.

"The assumed 90% theoretical sugar-to-fatty-alcohol conversion yield represents an upper-bound, literature-informed target consistent with near-theoretical yields reported for optimized microbial systems producing ethanol[1,2], and is intended to reflect a forward-looking scenario rather than current industrial performance."

[1]https://pubmed.ncbi.nlm.nih.gov/22888758/
[2]https://pubmed.ncbi.nlm.nih.gov/30568731/

4. The TEA mentions modeling of a product mixture, but provides no meaningful discussion of downstream separation and purification steps. Key information is missing regarding separation strategy, recovery efficiencies, and associated costs. DSP often dominates overall process economics and should be described explicitly.

This is a critical point, as the recovery and separation of individual fatty alcohols—given the product distribution in the fermentation broth (see Supplementary Figure 1b)—can be challenging when targeting industrial-grade purity. In this work, we assume centrifugation for cell mass removal, followed by decantation and distillation to recover a mixed fatty alcohol stream. We further assume that this mixture can be directly utilized as a diesel blendstock. All DSP details are included in the json file we have now provided for improved model transparency.

The reviewer is correct that additional downstream purification would be required to isolate individual alcohols for use in specialty chemicals or higher-value applications. Such upgrading would incur additional capital and energy costs, which are not included in the present analysis. This clarification has been added to the Methods section of the revised manuscript. See below text.

"Downstream processing was modeled assuming centrifugation for cell removal, followed by decantation and distillation to recover a mixed fatty alcohol stream suitable for use as a diesel blendstock; no further purification to isolate individual alcohols was included. Detailed separation assumptions, recovery efficiencies, and associated costs are provided in the accompanying Supplemental JSON flowsheet and deposited in the publicly accessible Project PISCES database (https://projectpisces.org/?page=database)."

5. Although biogas recovery from wastewater treatment is included for energy recovery, no quantitative energy balance is presented. The authors should quantify the fraction of total plant energy demand offset by biogas and discuss the implications for operating costs and overall process sustainability.

This is an important point. Biogas recovery from wastewater treatment offsets a substantial fraction of total plant energy demand. In the SOT scenario, it offsets ~44% of energy demand for the anaerobic case, while in the aerobic case it offsets only ~4%. The high offset in the anaerobic SOT scenario is due to unutilized xylose remaining in the wastewater, which contributes to the biogas yield. In contrast, the aerobic SOT case is very energy intensive, resulting in a smaller contribution from biogas. Under optimized conditions, when both glucose and xylose are utilized, biogas offsets ~21% in the anaerobic case and ~7% in the aerobic case. These contributions reduce reliance on purchased energy, lowering operating costs and enhancing overall process sustainability. We have included a bar chart showing the energy balance and contributions from biogas, cell mass, and purchased natural gas in the revised supplemental information (Supplementary Figure 3a).
